# Chromosome-level changes and genome elimination by manipulation of *CENH3* in carrot (*Daucus carota*)

**DOI:** 10.3389/fpls.2023.1294551

**Published:** 2023-11-15

**Authors:** Chandler M. Meyer, Irwin L. Goldman, Patrick J. Krysan

**Affiliations:** Department of Plant and Agroecosystem Sciences, University of Wisconsin-Madison, Madison, WI, United States

**Keywords:** CENH3, carrot, gene editing, tetraploid, doubled haploid

## Abstract

Hybrid cultivars are valuable in many crop species due to their high yield, uniformity, and other desirable traits. Doubled haploids, which have two identical sets of chromosomes, are valuable for hybrid breeding because they can be produced in one generation, in comparison to the multigenerational process typically used to produce inbred parents for hybrid production. One method to produce haploid plants is manipulation of centromeric histone H3 (*CENH3*). This method of producing haploids has so far been successful in *Arabidopsis*, maize (*Zea mays*), and wheat (*Triticum aestivum*). Here we describe modification of *CENH3* in carrot (*Daucus carot*a) to test for the ability of these modifications to induce uniparental genome elimination, which is the basis for haploid induction. Base editing was used to make *cenh3* mutant plants with amino acid substitutions in the region of *CENH3* encoding the histone fold domain. These *cenh3* mutant plants were then outcrossed with *CENH3* wild-type plants. Using PCR-based genotyping assays, we identified two candidates for genome elimination. One candidate was classified as a putative aneuploid plant in which chromosome 7 is in a single copy state. The other candidate was characterized as a putative tetraploid that was likely haploid during its genesis. Our results suggest that this putative tetraploid inherited all of its chromosomes from the *CENH3* wild-type parent and that the genome of the *cenh3* mutant plant was lost. This study provides evidence that modification of *CENH3* in carrot has the potential to induce genome elimination and ploidy changes in carrot.

## Introduction

Heterosis, also known as hybrid vigor, refers to the phenomenon whereby crosses between genetically distinct individuals result in progeny with superior performance when compared to the parental lines (Birchler et al., 2010). This phenomenon is most apparent when the two parents are highly homozygous. To produce highly inbred parental lines, plants can be self-pollinated or sib-mated for many generations in order to achieve desired levels of homozygosity, which is a time and resource intensive process. An alternative to inbreeding is the use of doubled haploids as parental lines for hybrid production (Forster and Thomas, 2005). The induction of haploid cells, followed by a subsequent doubling of the chromosomes, produces doubled haploids, which can function as “instantaneous inbred” lines and be produced in one generation. High levels of homozygosity can therefore be achieved in a much shorter timeframe compared to the inbreeding approach. This strategy significantly reduces the time and resources required to make hybrid varieties since it bypasses many generations of self- or sib-mating to create an inbred plant.

Established methods of haploid production include anther culture, ovule culture, and crossing with haploid inducing genotypes or species. In maize, doubled haploid lines are produced via an *in vivo* maternal haploid induction method, in which pollination is performed using pollen from inducer lines with specific genotypes (Chaikam et al., 2019). The gene that underlies this process in maize is *Matrilineal* (*MTL*), a patatin-like phospholipase expressed primarily in the pollen (Kelliher et al., 2017). Another method that can induce haploids in plants involves manipulation of centromeric histone H3 (*CENH3)*. In eukaryotes, CENH3 (CENP-A in mammals, CID in *Drosophila*) epigenetically specifies the location of the centromere (Mendiburo et al., 2011; Comai et al., 2017). The assembly of the kinetochore takes place at the centromere, an important process for proper segregation of chromosomes to daughter cells (Yu et al., 2000). Studies in *Arabidopsis thaliana (* Ravi and Chan, 2010; Karimi-Ashtiyani et al., 2015; Kuppu et al., 2015; Maheshwari et al., 2015; Kuppu et al., 2020; Marimuthu et al., 2021), maize (*Zea mays*) (Kelliher et al., 2016; Wang et al., 2021), and wheat (*Triticum aestivum*) *(* Lv et al., 2020), have shown that a cross between a *cenh3* mutant plant and a *CENH3* wild-type plant can lead to uniparental genome elimination, resulting in progeny that only inherit chromosomes of the wild-type parent.

Multiple approaches have been taken to manipulate *CENH3* in plants in an attempt to induce haploids. In one approach, transgenic plants in a *cenh3/cenh3* null background express a CENH3 protein in which the native N-terminal domain has been replaced with the tail of another histone variant and a fluorescent protein is fused to the N-terminus (Ravi and Chan, 2010; Kelliher et al., 2016). This approach has produced haploids in both *Arabidopsis* and maize. In another strategy, which has produced haploids in *Arabidopsis*, transgenic plants in a *cenh3/cenh3* null background expressed CENH3 proteins that contained amino acids substitutions or small deletions in the histone fold domain (Karimi-Ashtiyani et al., 2015; Kuppu et al., 2015; Kuppu et al., 2020). In a third approach, which produced haploids in maize, genome editing was used to produce plants with a heterozygous null mutation (*cenh3/CENH3*) in the endogenous copy of *CENH3* (Wang et al., 2021). Finally, haploids were produced in wheat through the use of genome editing to produce plants with restored frameshift mutations or deletions in the endogenous copy of *CENH3* (Lv et al., 2020).

Beyond maize and wheat, manipulation of *CENH3* leading to production of haploid progeny has not been reported for other crop plants. Carrot (*Daucus carota*) is an economically important vegetable crop that is a major contributor of vitamin A to the human diet (Simon et al., 2008). Hybrid cultivars are a cornerstone of carrot production due to their high yield and uniformity. The process of making carrot inbred lines usually involves six to ten generations of self-pollination or sib-mating to achieve desirable levels of homozygosity (Simon and Goldman, 2007). Since carrot is a biennial plant, this process is even more resource intensive, because it requires two seasons of growth to complete one life cycle. On the whole, it takes six to ten years, or 12 to 20 growth cycles, to make an inbred carrot plant. Therefore, the use of doubled haploids, which can be produced in a single generation, could significantly reduce the amount of time and resources to make carrot hybrids. In addition, the continued reliance on 3-way hybrids in carrot is an additional justification for the development of alternative methods of inbred development in carrot. Current methods for production of carrot haploids, which include *in vitro* culturing of ovules, anthers, and isolated microspores, are too inefficient and genotype dependent to be used for commercial production (Kiełkowska and Kiszczak, 2023). Therefore, a method for creating an efficient *in vivo* haploid inducer would be of great value for hybrid carrot production.

Manipulation of *CENH3* in carrot was previously described by Dunemann et al. (2019) and Dunemann et al. (2022). In these studies, the *cenh3* mutant plants tested were heterozygous and/or chimeric for mutations in *CENH3*. In that study, evidence was reported for the existence of a second copy of CENH3 in carrot, but it was not clear if this second copy was expressed. The production of haploids from crosses between the *cenh3* mutants and wild-type plants was not reported (Dunemann et al., 2019; Dunemann et al., 2022).

For this study, we used genome editing to make amino acid substitutions in the region of *CENH3* encoding the histone fold domain. We chose this strategy based on previous reports with *Arabidopsis* in which transgenic plants expressing a CENH3 protein with single or double amino acid substitutions in the histone fold domain produced haploids upon outcrossing with wild-type plants (Kuppu et al., 2015; Kuppu et al., 2020). Of the 38 single amino acid changes tested in those studies, 24 resulted in the induction of haploid progeny upon outcrossing. The rate of haploid induction varied depending on the particular amino acid substitution, however. Seven of the amino acid substitutions resulted in haploid induction rates >10%, with one amino acid substitution resulting in a haploid induction rate of 44%. Of the six double amino acid substitutions tested, three resulted in haploid induction rates >10%. Because the CENH3 histone fold domain is highly conserved across all plant species, including carrot (Kuppu et al., 2015), we were interested in determining if similar mutations in carrot would lead to genome elimination.

To explore if expression of variant CENH3 proteins can induce genome elimination and haploid induction in carrot, we used base editing to create *cenh3* mutant carrot lines expressing CENH3 proteins containing amino acid substitutions in the histone fold domain. Here we report our analysis of the progeny produced by crossing these *cenh3* mutant lines with *CENH3* wild-type plants. The results provide evidence that mutation of *CENH3* in carrot can lead to genome elimination and changes in ploidy.

## Materials and methods

### Plant material, protoplast isolation, transformation, and regeneration

Seeds from the carrot cultivar ‘Dolanka’ (W. Legutko, Żerków, Poland) were used as donor tissue for all gene editing experiments. Protoplast isolation, transformation, and regeneration were conducted as described in Meyer et al. (2022). Briefly, carrot protoplasts were prepared from *in vitro* grown two-week old carrot seedlings using enzymatic digestion of tissue followed by various washing steps. Protoplasts were then transfected with a base editing construct (STU-CBE1) (Meyer et al., 2022) via polyethylene glycol (PEG)-mediated transformation. A full DNA sequence map of STU-CBE1 is provided in **Supplemental File 1**. STU-CBE1 expresses a cytosine base editor that contains the following functional domains: APOBEC-3A cytosine deaminase, Cas9 D10A nickase, and Uracil DNA glycosylase inhibitor (UGI). Expression of the cytosine deaminase is driven by the cauliflower mosaic virus 35S promoter. STU-CBE1 is a single-transcript editing construct in which the guide RNA sequence is transcribed along with the cytosine deaminase from the 35S promoter. Following transformation, protoplasts were embedded in a thin alginate layer and cultured in protoplast culture medium (CPP) (Dirks et al., 1996; Grzebelus et al., 2012). During culture, macro-colonies, pro-embryonic masses (PEM), and somatic embryos formed. After approximately 8-9 weeks of culture in CPP medium, macro-colonies, PEM, and somatic embryos were released from the alginate layer by incubation in a sodium citrate solution (Meyer et al., 2022). The macrocolonies, PEM and somatic embryos were then re-suspended in CPPD (modified version of CPP medium) (Dirks et al., 1996) solution and aliquoted onto petri dishes containing filter paper resting on the surface of regeneration (R) medium (Dirks et al., 1996). Somatic embryos, PEM and macrocolonies were periodically transferred to new Petri dishes containing R medium without filter paper for continued growth. When plants growing on R medium had produced at least 2.5 cm of shoot growth and had well-developed roots, they were transferred to potting soil and covered with a humidity dome to begin *ex vitro* acclimatization. Using a PCR-based genotyping method, we identified 50 plants with homozygous or bi-allelic mutations in *CENH3* (Meyer et al., 2022). The specific DNA sequence details of these *cenh3* mutant lines were previously described (Meyer et al., 2022). Nineteen of these *cenh3* mutant plants were used for crosses with *CENH3* wild-type plants to test for genome elimination and haploid induction.

### DNA extraction from seedling tissue for genotyping

For PCR-based genotyping, DNA was extracted from young seedling tissue by cell lysis using the Bullet Blender tissue homogenizer (Next Advance, USA, catalog # BBY24M). To extract DNA, approximately 10 mg of leaf tissue was placed into a 1.5 ml microcentrifuge tube containing a 3.2 mm diameter stainless steel ball and 250 µl of Lysis Buffer (20 mM Tris (pH 8), 2.5 mM EDTA, 25 mM NaCl, 0.05% (w/v) sodium dodecyl sulfate). The microcentrifuge tube was then placed in the Bullet Blender tissue homogenizer for 1 min at speed = 10. The solution was then centrifuged at 17,000 × g for 2 min. 5 µL of crude extract was collected and placed into 120 µL of water to produce the final DNA solution for use in PCR.

### PCR Allele competitive extension-based genotyping assay to characterize progeny

For the PACE™ assay, allele-specific primers with different fluorescent tags (FAM or HEX) were designed to discriminate between the *cenh3*-edited allele and the *CENH3* wild-type allele. Two allele-specific primers were designed, one to amplify the *cenh3*-edited alleles and one to amply the *CENH3* wild-type allele. The sgRNA1 target site in *CENH3* was amplified using PACE® 2.0 Genotyping Master Mix (3CR Bioscience, UK) and a SNP-specific assay mix comprising two allele-specific forward primers, 5’-GAAGGTGACCAAGTTCATGCTATCTCACGAAGAGCCACGGTAC-3’ (FAM) and 5’ -GAAGGTCGGAGTCAACGGATTAATCTCACGAAGAGCCACGGTAT 3’ (HEX) and one common reverse primer, 5’ CAACAGCGAAAGCCCCACAGATTTA-3’. Progeny were classified as self-pollinations, F1s, or candidates for genome elimination based on the fluorescence generated during these end-point genotyping reactions. Progeny were classified as self-pollinations if the sample displayed only HEX fluorescence. Progeny were classified as F_1_ progeny if the sample displayed both FAM and HEX in approximately equal amounts. Progeny were classified as candidates for genome elimination if the sample displayed only FAM fluorescence.

### Sanger-based genotyping assay to characterize progeny

For the Sanger sequencing assay, the sgRNA2 target site in *CENH3* was amplified using Forget-Me-Not™ EvaGreen® qPCR Master Mix (Biotium, USA, catalog # 31046) and the following pair of PCR primers 5′-AGTACTGCTACCCCGAGTAAGTC-3′ and 5′-GCGATAACTTACAGTGCGGATAAAC-3′. Amplified PCR products were sequenced using a Sanger sequencing service (Genewiz LLC, USA). Sanger sequencing chromatograms were manually inspected to determine the nucleotide sequence at the *CENH3* target site. Progeny were classified as self-pollinations if they were homozygous for the *cenh3*-edited allele. Progeny were classified as F_1_ progeny if they were heterozygous for the *cenh3*-edited and *CENH3* wild-type sequence. Progeny were classified as candidates for genome elimination if they were homozygous for the wild-type *CENH3* sequence.

### DNA quantification and library preparation for whole genome re-sequencing

Library construction, genome sequencing, and raw data processing were conducted by Bejing Genomics Institute (BGI). For genomic library construction, the library was prepared using the DNBseq™ Normal DNA library construction method (BGI). Libraries with an insert size of ca. 300 bp were constructed. Amplification of the ligation products was done by PCR amplification. The libraries were then sequenced on the DNBseq™ sequencing platform (Beijing Genomics Institute) to generate 150 bp paired-end reads. On average, 138,734,598 150 bp raw reads were generated per sample. Adapter trimming of the raw reads, low quality read trimming, and contiguous N bases trimming were performed using the SOAPnuke tool (Chen et al., 2017). Reads were then aligned to the *D. Carota* V2.0 reference genome (Iorizzo et al., 2016) using the Burrow-Wheeler Aligner (Li and Durbin, 2009). On average, 134,961,912 mapping reads were generated per sample. The mean depth per sample was 46.7 and the average coverage (>= 1X) was 86.8% (**Supplementary Table S2**).

### Dosage analysis

Mapped sequencing reads were obtained from sequence alignment map (SAM) files generated by the whole genome sequencing process. Reads were filtered so as to only retain sequence reads in which the reads were paired and mapped in the proper pair, the paired reads aligned to the same contig, and the read mapped to one unique position (FLAG = 163 or 83 or 99 or 147, RNEXT = ‘=", MAPQ=60). Following filtering, each of the nine chromosomes was *in silico* divided into 250 kb-sized bins. Then, mapped sequencing reads were assigned to each bin based on the mid position boundary location of the read. The total number of mapped reads in each bin was recorded to obtain the raw read depth value. The raw read depth value was then divided by the total number of reads for that chromosome to obtain the read depth percentage for each bin. The read depth percentage was normalized by dividing the read depth percentage value by the average read depth percentage value of the four wild-type control samples. The normalized read depth value was then multiplied by two to represent a diploid chromosome copy number of two and plotted.

### Genome wide estimation of heterozygosity

Sequence alignment map (SAM) files were used to identify SNPs using the bcftools package version v1.16 (Li, 2011). The *D. Carota* V2.0 reference genome (Iorizzo et al., 2016) was used as a reference. SNPs were called using the bcftools mpileup command with the following parameters: skip indels, max depth of 100 and minimum mapping quality of 55 (bcftools mpileup -I -d 100 -q 55). Post-filtering of SNPs was then performed using the bcftools view command to remove SNPs with a mapping quality below 140, a depth greater than 50, and a SCBZ score greater than 0 (bcftools view -e 'QUAL <= 140 || DP > 50 || SCBZ > 0'). Then, homozygous SNPs were removed and therefore only heterozygous SNPs were retained. For the SNP analysis, each of the nine chromosomes was in silico divided into non-overlapping 250 kbp-sized bins. Each SNP was assigned to each bin based on the mapped position of the SNP. The total number of heterozygous SNPs was recorded to obtain the total number of heterozygous SNPs for each chromosomal bin.

### Flow cytometry to estimate nuclear DNA content

Nuclear DNA content was measured by flow cytometry using young leaf tissue from mature soil-grown plants that were 4 to 8 weeks old. Leaf tissue of a carrot diploid ‘Dolanka’ (2n=2x=18) plant was used as a reference standard. For isolation of nuclei from leaf tissue, a total of 50 mg of leaf tissues was placed into a chilled 100 mm glass petri dish containing 700 µl of lysis buffer (10mM Tris (pH 7.0), 2 mM MgCl2, 50 mM sodium chloride, 1% (w/v) polyvinylpyrrolidone, 0.1% (v/v) TRITON X-100) (Kiełkowska and Adamus, 2010). The tissue was chopped with a razor blade into fine pieces for approximately 2 min. After chopping, the homogenate was filtered through a 70 µm cell strainer to remove large debris. An equal volume of FxCycle™ PI/RNase Staining Solution (Thermo Scientific, USA, catalog # F10797) was added to the homogenate for staining and samples were stored on ice. Samples were incubated on ice at least 20 min before measurements were taken. The DNA content was measured using an Attune™ NxT Flow Cytometer (Thermo Scientfic, USA). To determine the DNA content of lines CM21 and CM23, two different methods of comparison were used. For the first method, leaf tissue of CM21 or CM23 and leaf tissue of the wild-type plant were placed in separate petri dishes for the extraction and staining of nuclei, with each dish containing a total of 50 mg of leaf tissue. For the second method, equal amounts of leaf tissue CM21 or CM23 and leaf tissue of the wild-type plant were placed in the same petri dishes for the extraction and staining of nuclei. The DNA content of CM21 or CM23 was determined by comparing the median value of the PI fluorescence peak of the nuclei of that sample to the median value of the PI fluorescence peak of the nuclei of the control wild-type diploid plant.

### Identification of parental lineage of the mitochondrial genomes of CM21 and CM23

To identify the parental origin of the mitochondrial genome of CM21 and CM23, the whole genome sequencing data of the two parental lines, RP31 and W255-02 for CM21, and RP23 and SW34 for CM23, were inspected manually using the NCBI Genome Workbench (Kuznetsov and Bollin, 2021) to identify a location in the mitochondrial genome in which there were polymorphisms between the parental lines. A forward primer named NAD7-F1 5’- CATAGCGATCTCCTCTGGTAC-3’ and a reverse primer named NAD7-R1 5’- AGCTCGCCTTCTTGTTATCCA-3’ were used to amplify the region. Amplified PCR products were sequenced using a Sanger sequencing service (Genewiz LLC, USA). The sequences from the FASTA files of the two crossing partners and the progeny were aligned using the multiple sequence alignment program ‘Clustal Omega’ (Sievers and Higgins, 2014. The trace files containing the sequencing chromatograms were further analyzed manually to validate the presence of each polymorphism and identify if the polymorphisms were in the homozygous or heterozygous state (**Supplementary Table S1**).

### Identification of parental lineage of the nuclear genome of CM21

To identify the parental origin of the nuclear genome of line CM21, the whole genome sequencing data of the two parental lines, RP31 and W255-02, was inspected manually using the NCBI Genome Workbench (Kuznetsov and Bollin, 2021) to identify locations in the nuclear genomes in which there were polymorphisms between the two parental lines. Using this analysis, eighteen locations in the nuclear genome were selected for PCR-based genotyping, two locations each chromosome (**Supplementary Table S2**). For each location in the genome, a forward primer and a reverse primer were designed to amplify the region (**Supplementary Table S3**). Amplified PCR products were sequenced using a Sanger sequencing service (Genewiz LLC, USA). The sequences from the FASTA files of CM21, RP31, and W255-02 were aligned using the multiple sequence alignment program ‘Clustal Omega’ (Sievers and Higgins, 2014). The trace files containing the sequencing chromatograms were further analyzed manually to validate the presence of each polymorphism and identify if the polymorphisms were in the homozygous or heterozygous state (**Supplementary Table S4**).

## Results

### Creation of cenh3 mutant lines

**Figure 1:**
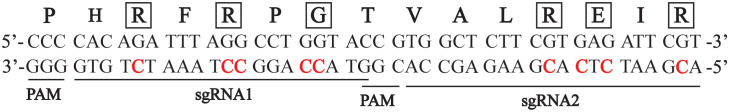
Sequence of the carrot *CENH3* genomic region used as the target site for gene editing. The sequence is located in the fourth exon of the *CENH3* genomic region. Locations of the two sgRNAs used are indicated. The amino acid sequence of the CENH3 protein is indicated above the DNA sequence. Cytosines that were targeted are indicated in red, and corresponding amino acids impacted by mutation of those sites are indicated outlines with boxes.

To test if a carrot plant expressing a variant CENH3 protein can function as a haploid inducer, we created gene-edited carrot plants with amino acid substitutions in the region of *CENH3* encoding the CENH3 histone fold domain. These mutations in *CENH3* were produced using a cytosine base editor (STU-CBE1) (Meyer et al., 2022). Two sgRNAs, sgRNA1 and sgRNA2, were designed to target a region in the fourth exon of *CENH3* (**Figure 1**). This region was chosen for two reasons: first, in studies in *Arabidopsis*, amino acid substitutions in this region resulted in haploid induction upon outcrossing to wild-type plants (Kuppu et al., 2015; Kuppu et al., 2020), and second, this region is highly conserved among dicots and monocots (Kuppu et al., 2015). Using a protoplast transformation and regeneration method described in Meyer et al. (2022), we produced gene-edited carrot plants with homozygous and/or heterozygous missense mutations in the region of *CENH3* encoding the histone fold domain. Nineteen of these *cenh3* mutant plants were then crossed with *CENH3* wild-type plants to test for genome elimination (**Table 1**). The cenh3 mutations present in these nineteen lines were grouped into 5 categories based on the specific amino acid changes present in CENH3. Two of these categories had single amino acid changes, one had two amino acid changes, and two had three amino acid changes to the CENH3 coding sequence (**Table 2**). The E58K and G52N amino acid changes present in the lines carrying a single amino acid substitution correspond to mutations that have been shown to cause haploid induction in Arabidopsis (Kuppu et al., 2015; Kuppu et al., 2020).

**Table 1:** *cenh3* mutant carrot lines used in crosses with CENH3 wild-type plants to test for genome elimination.

Line	*cenh3* mutant category	Allele 1	Allele
RP32	1	G52N	G52N
RP71	1	G52N	G52N
RP156	1	G52N	G52N
RP62	2	R50K, G52N	R50K, G52N, R48K
RP68	2	R50K, G52N	R50K, G52N, R48K
RP165	2	R50K, G52N	R50K, G52N, R48K
RP25	3	E58K	E58K
RP100	3	E58K	E58K
RP104	3	E58K	E58K
RP114	3	E58K	E58K
RP165	3	E58K	E58K
RP23	4	E58K	E58K, R60H
RP65	4	E58K	E58K, R60H
RP31	4	E58K	E58K, R60H
RP14	4	E58K	E58K, R60H
RP67	4	E58K	E58K, R60H
RP61	5	E58K	E57H, E58K, R60H
RP82	5	E58K	E57H, E58K, R60H
RP158	5	E58K	E57H, E58K, R60H

Sanger sequencing was used to determine the DNA sequences of the target window of the CENH3 gene in each mutant line. The amino acid sequences of the CENH3 proteins encoded by each cenh3 mutant plant were then deduced. Both homozygous and biallelic mutations were recovered. The predicted amino acid changes for each allele are listed. Overall, 19 cenh3 mutant lines were created with multiple independent lines encoding each CENH3-variant protein.

**Table 2:** Analysis of genetic crosses between *cenh3* mutant plants and *CENH3* wild-type (WT) plants.

cenh3 mutant line	Allele1	Allele 2	Germination rate (%)	Total Progeny screened	F_1_ Progeny	Candidates	Self-pollination rate in surveyed progeny (%)
RP32	G52N	G52N	55.5	40	40	0	0.0
RP71	G52N	G52N	84.5	150	142	0	5.3
RP156	G52N	G52N	77.1	109	99	0	9.2
RP62	R50K,G52N	R50K,G52N,R48K	75	143	91	0	36.4
RP68	R50K,G52N	R50K,G52N,R48K	71.4	109	15	0	86.2
RP165	R50K,G52N	R50K,G52N,R48K	69.6	75	0	0	100.0
RP25	E58K	E58K	25.0	23	23	0	0.0
RP100	E58K	E58K	NT	n/a	n/a	0	n/a
RP104	E58K	E58K	NT	n/a	n/a	0	n/a
RP114	E58K	E58K	20.8	26	2	0	92.0
RP165	E58K	E58K	13.5	11	0	0	100.0
RP23	E58K	E58K,R60H	23.1	116	24	1	78.9
RP65	E58K	E58K,R60H	NT	n/a	n/a	0	n/a
RP31	E58K	E58K,R60H	15.2	44	15	1	65.1
RP14	E58K	E58K,R60H	15.6	9	7	0	22.2
RP67	E58K	E58K,R60H	9.2	20	18	0	10.0
RP61	E58K	E57H,E58K,R60H	1.8	2	0	0	100.0
RP82	E58K	E57H,E58K,R60H	0.7	1	0	0	100.0
RP158	E58K	E57H,E58K,R60H	4.1	3	0	0	100.0

A total of 772 progeny were screened. Two progeny were identified as candidates for genome elimination based on their genotype at the CENH3 locus. One progeny came from a cross between RP23 and CENH3 wild-type line SW34 and the other came from a cross between RP31 and CENH3 wild-type line W255-02. NT indicates progeny were not tested for germination.

### Screening progeny for evidence of genome elimination

**Figure 2:**
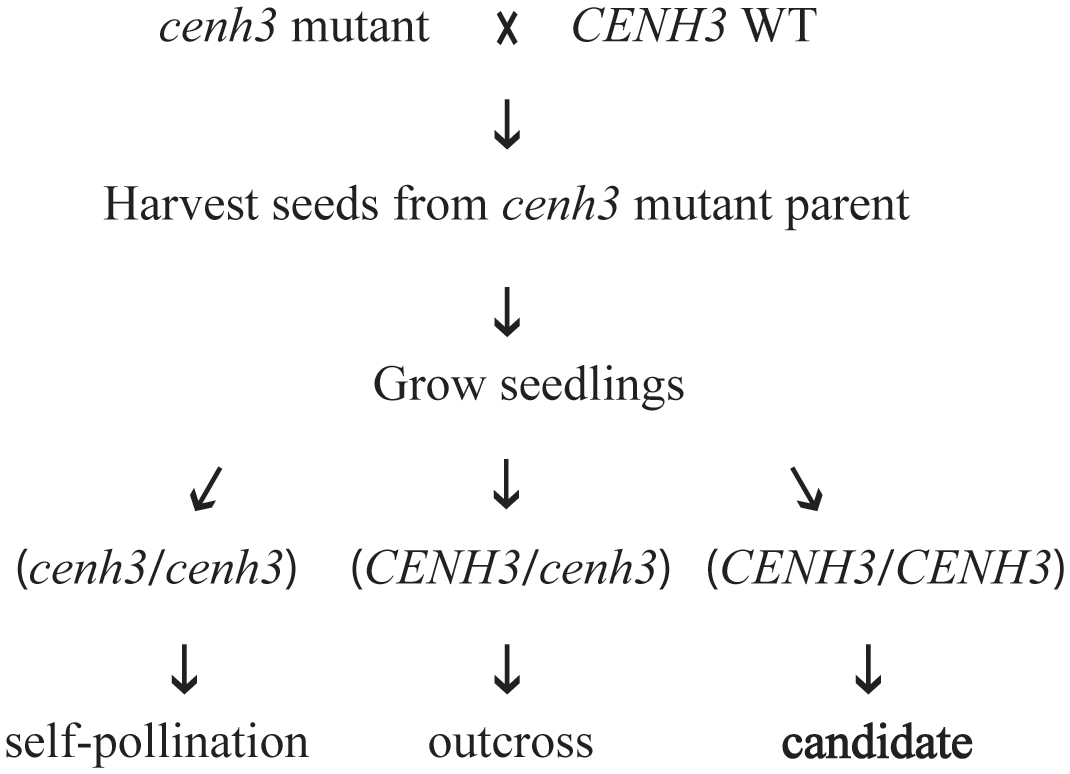
Schematic representation of test crosses between *cenh3* mutant plants and *CENH3* wild-type (WT) plants. Progeny produced by these crosses were tested for evidence of genome elimination. Only progeny for which the *cenh3* mutant plant was the female parent were analyzed. The DNA sequence of the exon 4 region of *CENH3* was determined for each seedling, and plants were classified as derived from self-pollination of the *cenh3* mutant parent (*cenh3*/*cenh3*), derived from cross-pollination (*CENH3*/*cenh3*), or as candidates for genome elimination derived from cross-pollination (*CENH3*).

In studies performed in *Arabidopsis*, haploid induction was strongest when the plant expressing the variant form of CENH3 was used as the female parent in crosses with a *CENH3* wild-type plant (Ravi and Chan, 2010; Karimi-Ashtiyani et al., 2015; Kuppu et al., 2020). Therefore, we focused our progeny testing on seed collected from the *cenh3* mutant plant. Our first step in screening progeny for the presence of genome elimination was to genotype the region of *CENH3* that was modified in the *cenh3* mutant parents. If genome elimination of the maternal chromosomes occurred, the progeny would only contain the wild-type allele from the wild-type parent. We therefore used either PCR Allele Competitive Extension (PACE™) (von Maydell, 2023) or Sanger sequencing to genotype this region of *CENH3* in progeny (**Figure 2**). The pollen donors in these crosses were homozygous for the wild-type *CENH3* sequence. Candidates for genome elimination were identified among the progeny produced by the *cenh3* mutant parent as seedlings that appeared homozygous for the wild-type *CENH3* sequence. These assays were also used to identify progeny that were the result of self-fertilization of the *cenh3* mutant parent (*cenh3/cenh3*) as well as progeny that were the result of cross-pollination (*CENH3/cenh3*). There did not appear to be any major differences between the overall growth and development of the *cenh3* mutant plants and wild-type. Among the progeny screened for evidence of genome elimination, we did observe a range of phenotypic variation in terms of seedling size and growth rate, but we did not attempt to correlate seedling phenotype with genotype for this study.

Using these assays, we screened a total of 855 progeny. Of the progeny screened, 476 were determined to be produced by outcrossing and therefore classified as F_1_ progney. Of these F_1_ progeny, we identified two plants, CM21 and CM23, that only inherited the *CENH3* wild-type allele at this locus (**Table 2**). Both of these progeny came from a cross with a *cenh3* mutant parent that was homozygous for two C to T transitions and heterozygous for a third. Therefore, there are two alleles of *cenh3* in this mutant parent. One allele encodes a CENH3 protein with an E58K amino acid substitution, and the other allele encodes a protein with E58K and R60H amino acid substitutions.

To confirm that the progeny CM21 and CM23 were produced by the *cenh3* mutant parent, manual inspection of the mitochondrial genome sequence using NCBI Genome Workbench (Kuznetsov and Bollin, 2021) was used to identify a region in the mitochondrial genome in which there were polymorphisms between the two parental crossing partners. Sanger sequencing of this region confirmed that, for both progeny, the *cenh3* mutant plant was the maternal parent (**Supplementary Table S1**).

### Evidence of aneuploidy or chromosomal abnormalities in line CM23

In previous studies on CENH3-based haploid induction in *Arabidopsis and* maize, aneuploidy and other chromosomal abnormalities are a common outcome (Kuppu et al., 2015; Tan et al., 2015; Kelliher et al., 2016). Therefore, the absence of the *cenh3* mutant allele in the candidate plants at a region in chromosome 7 could be explained by aneuploidy or chromosomal abnormalities. For this reason, we further investigated if the entirety of chromosome 7 had been lost and if there were chromosomal abnormalities in other parts of the genome. To do this, we performed a dosage analysis using whole genome sequencing data. Whole genome sequencing was performed on the progeny CM21 and CM23, as well as on four wild-type plants grown in soil that were used as controls. On average, 135,386,818 raw sequencing reads were generated per sample. The raw reads were then mapped to the *D. Carota* V2.0 reference genome (Iorizzo et al., 2016). The average depth per sample was 45.8 and the average coverage (>1X) was 86.8% (**Supplementary Table S5**).

**Figure 3:**
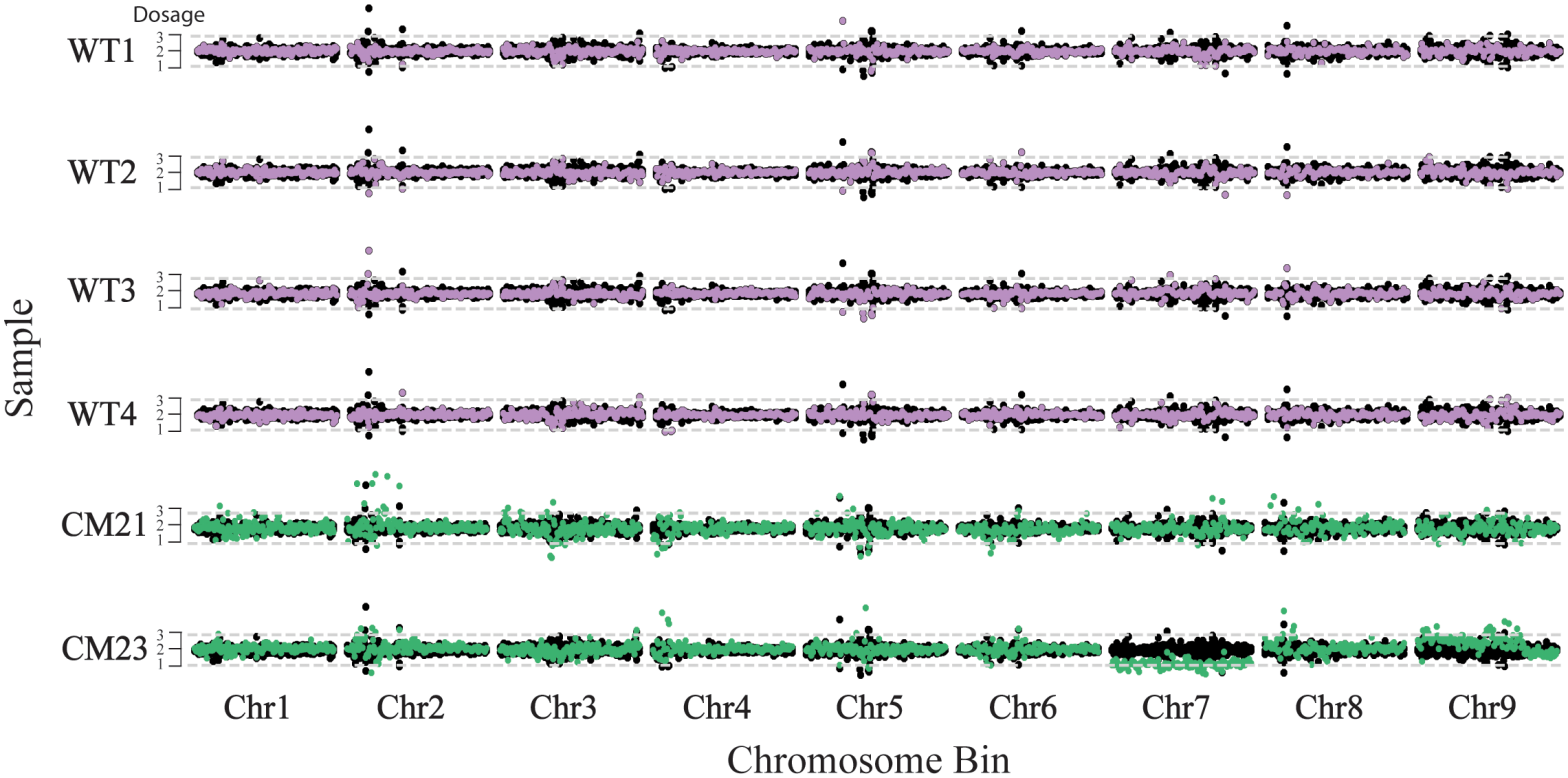
Chromosome dosage analysis to detect aneuploidy in lines CM21 and CM23. For each sample, the normalized read count is plotted on the y-axis vs the 250 kpb-sized chromosome bins on the x-axis. Each horizontal track represents the nine chromosomes of an individual plant. To represent the expected dosage variation in a normal genome, the normalized read count of four WT plants are consecutively overlaid on top of each other and plotted in black. The normalized read depth of each sample is overlaid on top and plotted in either purple (WT) or green (candidate). A value of two represents the expected chromosome copy number of a diploid plant. Chromosomal segments containing bins with values of approximately one would indicate potential chromosome loss and segments containing bins with values of approximately three or more would indicate potential chromosome duplication.

Dosage analysis was used to estimate the relative copy number of the chromosomes in these plants. For this analysis, the chromosomes of each individual were *in silico* divided into 250 kbp-sized bins and each sequence read was assigned to a bin based on the mapped position of that read. To standardize read counts, the number of reads for each bin was divided by the total number of reads across all bins of the sample, giving a percent reads value for each bin. This percent reads value was then normalized by dividing the percent read value for each bin by the average percent read value of the corresponding bin of the four wild-type controls. This normalized ratio was then multiplied by two to represent a chromosome copy number of two of a diploid plant. As seen in **Figure 3**, the entirety of chromosome 7 of CM23 has a lower dosage compared to the wild-type controls, with the values oscillating around 1. In addition, a large portion of chromosome 9 of CM23 has a slight increase in dosage in which the chromosomal bins oscillate somewhere between a value of 2 and 3. CM23 appears to be an aneuploid in which elimination of chromosome 7 has occurred. The slight increase in dosage on a large segment of chromosome 9 could be evidence of another chromosomal abnormality. The chromosomes of CM21 did not display any large-scale increase or decrease in relative dosage, and therefore there was no evidence of aneuploidy in CM21 (**Figure 3**). Because the method used for this analysis measures the dosage the chromosomes relative to each other, it is not possible to detect whole-genome duplication or haploidy using this approach.

### Increased nuclear DNA content in line CM21

In studies on CENH3-mediated haploid induction in *Arabidopsis*, it was observed that chromosome elimination occurs during the first few embryonic mitotic divisions (Marimuthu et al., 2021). If all the chromosomes inherited from one parent are eliminated from the nucleus of the zygote, then the progeny becomes a haploid plant with half the DNA content of a normal diploid. To search for evidence of genome elimination, we used flow cytometry to measure the DNA content of CM21 by comparing the median value of the propidium iodide (PI) fluorescence peak of nuclei extracted from CM21 to the median value of the PI fluorescence peak of nuclei extracted from a normal diploid carrot plant. For this analysis, we used two different methods to compare line CM21 to the reference standard since the amount of PI fluorescence exhibited by nuclei is influenced by sample prep and density of nuclei. For one method, we isolated and stained nuclei from line CM21 and the wild-type plant separately. For the other method, we mixed the leaf tissue of line CM21 and the wild-type plant together before isolating and staining nuclei.

**Figure 4:**
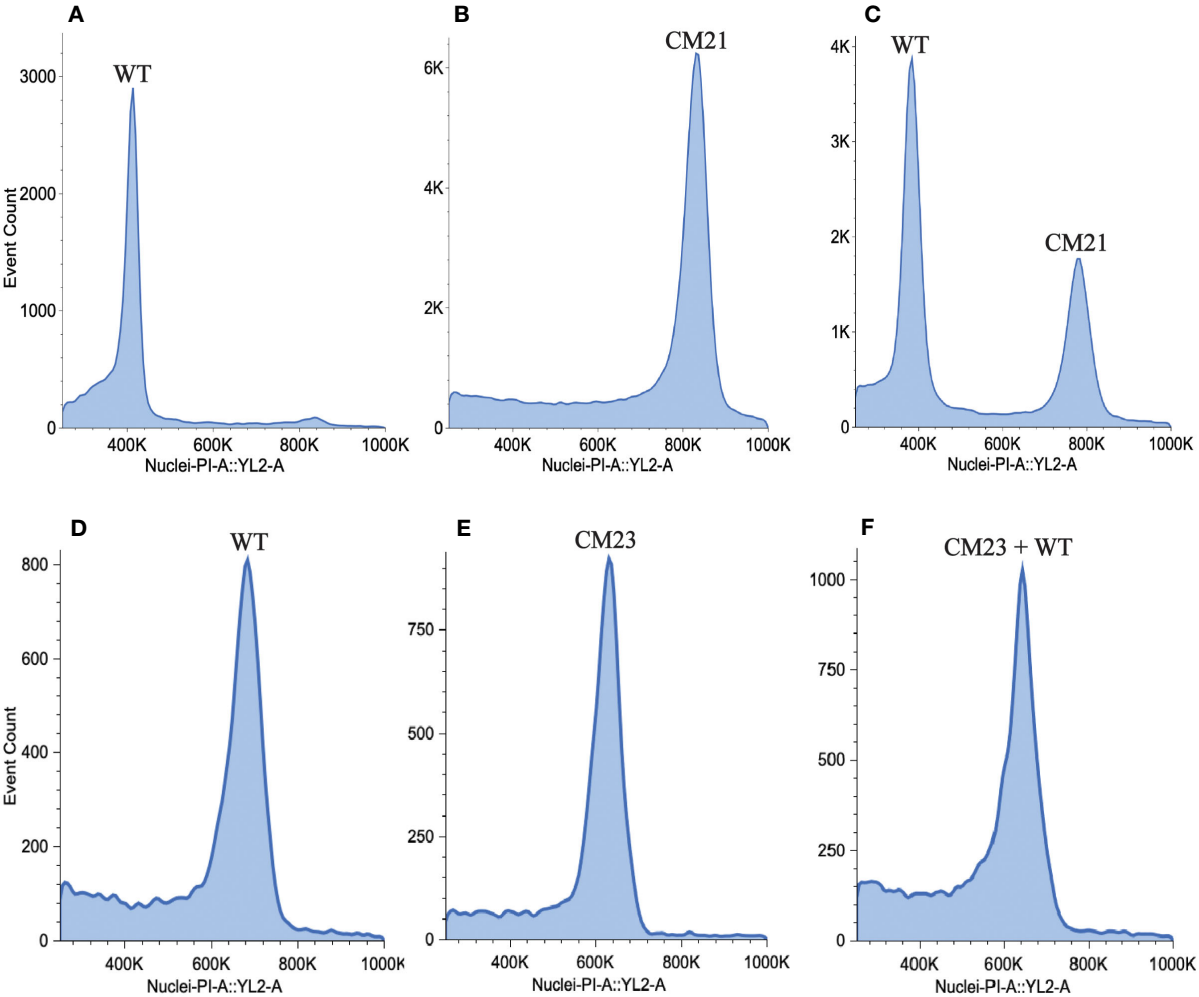
Estimation of nuclear DNA content of lines CM21 and CM23. To estimate nuclear DNA content, flow cytometry analysis was performed on line CM21 and CM23. A diploid WT carrot plant was used as the reference standard. Histograms show propidium iodide (PI) fluorescence of nuclei isolated from **(A)** Line CM21 only **(B)** WT only **(C)** WT mixed with line CM21 **(D)** line CM23 only **(E)** WT only **(F)** WT mixed with line CM23. The median fluorescence value of CM21 is approximately twice the value of the median fluorescence value of the diploid control indicating that CM21 is likely a tetraploid. The median fluorescence value of CM23 is approximately equal to the median fluorescence value of the diploid control, indicating that the nuclear DNA content of CM23 is similar to that of the diploid control.

As seen in **Figures 4A, B**, the median value of the PI fluorescence peak of nuclei from CM21 is approximately twice the value as that of the wild-type plant (**Supplementary Table S6**). When the samples were mixed, there were two distinct peaks, with the peak of CM21 approximately twice the value of the wild-type peak (**Figure 4C**). These data indicate that CM21 has approximately twice the amount of DNA content as the diploid control and therefore is likely a tetraploid. We also performed this analysis on CM23 to determine its DNA content. As seen in **Figures 4D, E**, the median value of the PI fluorescence peak of nuclei from CM23 is approximately the same as that of the wild-type plant. When the samples were mixed, there was only one peak, indicating that the DNA content of line CM23 was very similar to that of the wild-type plant (**Figure 4F**). Therefore, CM23 is likely a plant with two sets of chromosomes in which chromosome 7 is in a single copy state and chromosome 9 is either shattered or has undergone partial duplication.

### Greatly reduced heterozygosity in line CM21

Our observation that line CM21 has twice the nuclear DNA content of a wild-type diploid carrot raises questions about the origin of the extra DNA present in this line. To investigate this question we began by using SNP analysis to estimate the level of heterozygosity in the genome of CM21. The rationale for this experiment was to determine if the multiple copies of each chromosome present in CM21 are derived from a single parent or from both of the parents involved in the cross that produced CM21. If the chromosomes all come from one parent, then we would expect no heterozygosity to be present in the genome of CM21. This outcome could be produced if genome elimination occurred in the initial zygote, followed by two rounds of spontaneous chromosome duplication. By contrast, if the chromosomes in CM21 were derived from both parents, then one would expect substantial heterozygosity throughout the genome since there are many polymorphisms between the two parental lines used for the cross that produced CM21.

**Figure 5:**
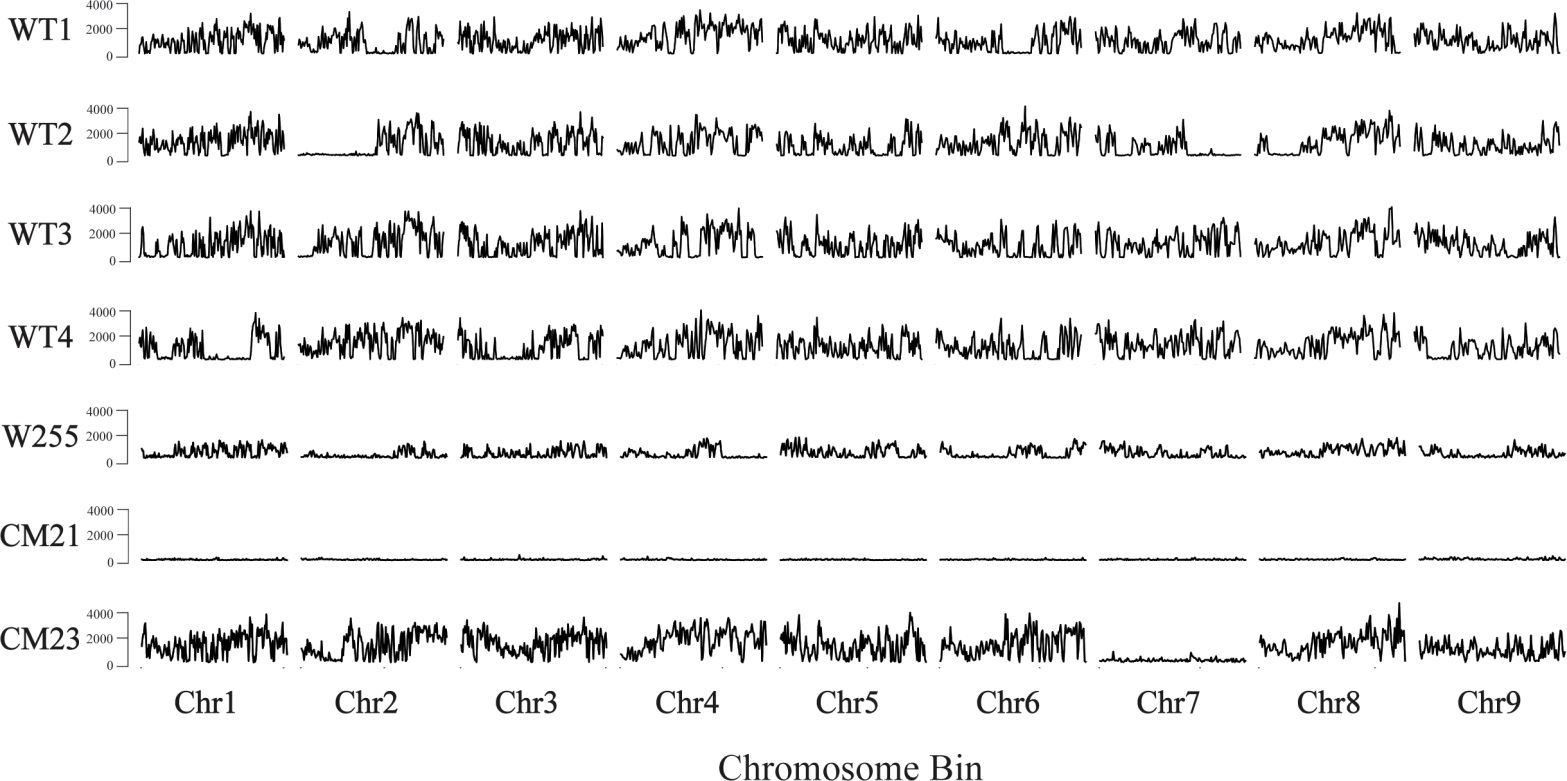
Genome wide estimation of heterozygosity in lines CM21 and CM23. For each sample, the number of heterozygous SNPs is plotted on the y-axis vs the 250 kpb-sized chromosome bins on the x-axis. Each horizontal track represents the nine chromosomes of an individual plant. Data for four WT ‘Dolanka’ plants and the inbred line ‘W255’ are shown as controls. All of the chromosomes of progeny line CM21 contain a substantial reduction in heterozygosity compared to the four WT plants and the inbred line, W255. For progeny line CM23, chromosomes 1-6, 8, and 9 appear to have similar levels of heterozygosity compared to the four WT plants and the inbred line, W255. However, the entirety of chromosome 7 of CM23 has a substantial reduction in heterozygosity.

To quantify heterozygosity, we used the whole genome sequencing data that was used for the dosage analysis described above. In addition, we also used previously collected whole genome sequencing data from a carrot inbred line ‘W255’ (Goldman, 1996) as a control. SNPs were identified using the v1.16 of the BCFtools package (Li, 2011; Danecek et al., 2021). After calling SNPs, the SNPs were filtered to discard the homozygous SNPs so that only the heterozygous SNPs were used for the analysis. Then, the distribution of heterozygosity across each chromosome for each plant was quantified by counting the total number of heterozygous SNPs in each 250 kbp-sized chromosomal bin. For this analysis, each chromosome of each individual was *in silico* divided into 250 kbp-sized bins and each SNP was assigned to a bin based on the mapped position of that SNP. The total number of heterozygous SNPs that fell into each bin was counted (**Figure 5**).

As seen in **Figure 5**, the amount of heterozygosity for each of the four wild-type controls varies across each chromosome. In some cases, certain sections of a chromosome contained large reductions in heterozygosity, which would be expected in an open pollinated population where self-fertilization sometimes occurs. The inbred plant ‘W255’ exhibited a lower overall level of heterozygosity compared to the wild-type plants, which is to be expected with a plant that was derived from a population which was created by multiple generations of self-pollination and sib-mating. CM23 appears to have a similar level of heterozygosity as the wild-type plants for every chromosome except chromosome 7. In CM23, there is a large reduction in heterozygosity across the entirety of chromosome 7, which is consistent with a model of whole chromosome loss at that chromosome. In the case of CM21, there is a substantial reduction in heterozygosity across all chromosomes when compared to the four wild-type plants and the inbred plant. This result is consistent with whole genome elimination of one of the parental genomes occurring during the genesis of CM21.

### Uniparental inheritance of the chromosomes in line CM21

The dosage analysis described above showed that there is no apparent aneuploidy in line CM21, and the genome-wide heterozygosity analysis indicated extremely low heterozygosity across all chromosomes in line CM21. One model that would explain these results is that genome elimination occurred during the formation of CM21 whereby the chromosomes of line CM21 originated from only one of the parents of the cross that produced the line. In previous studies in *Arabidopsis* and maize, when genome elimination occurs, it is always the chromosomes of the *cenh3* mutant parent that are lost (Ravi and Chan, 2010; Kelliher et al., 2016; Marimuthu et al., 2021). Therefore, our hypothesis was that the four copies of each chromosome present in line CM21 originated from the CENH3 wild-type parent (W255-02) and that the genome of the *cenh3* mutant parent (RP31) had been lost.

To test this hypothesis, we used PCR genotyping to identify the parental origin of the chromosomes present in CM21. To begin with, we performed manual inspection of the whole genome sequencing data of the CM21 parental lines using NCBI Genome Workbench (Kuznetsov and Bollin, 2021) and identified two regions on each of the nine chromosomes containing polymorphisms between the two parents, RP31 and W255-02, for a total of 18 regions. Next, we designed PCR primers to amplify these 18 regions and used Sanger sequencing to determine the DNA sequence of these regions in the two parental lines and line CM21 (**Supplementary Table S4**). If line CM21 only inherited the chromosomes of RP31, it would be homozygous for the RP31 haplotype and if it only inherited the chromosomes of W255-02, it would be homozygous for the W255-02 haplotype. The sequencing data revealed that, at all 18 regions, the sequence of CM21 was an exact match to the sequence of W255-02, the CENH3 wild-type parent from the cross that produced CM21. In addition, in all 18 regions, CM21 was polymorphic from RP31, the *cenh3* mutant parent. These data are consistent with a model in which CM21 inherited all of its chromosomes from the *CENH3* wild-type parent W255-02.

## Discussion

The ability to efficiently induce the formation of haploid plants has value for both basic biological studies and as a breeding tool to rapidly create inbred plants for hybrid variety production. In this study, we demonstrate that mutations in carrot *CENH3* can lead to the loss of one parental chromosome set and the production of aneuploidy when those *cenh3* mutants are crossed with plants carrying a wild-type *CENH3*. In previous studies on CENH3-mediated haploid induction, multiple strategies were used to manipulate *CENH3* in an attempt to induce haploids. So far, manipulation of *CENH3* has produced haploids in *Arabidopsis*, maize, and wheat. However, CENH3-mediated haploid induction has not been applied or proven successful in other crop plants. Studies by Dunemann et al. (2019) and Dunemann et al. (2022) involved carrot plants with heterozygous and/or chimeric mutations in *CENH3*. However, haploids were not observed among the progeny of crosses between these *cenh3* mutants and wild-type plants.

In this study we explored if single amino acid substitutions in the histone fold domain of the endogenous copy of carrot *CENH3* could lead to uniparental genome elimination in the progeny. In our experiments, *cenh3* mutant plants were crossed with *CENH3*-wild type plants, and the progeny were analyzed. One of the crosses between a *cenh3* mutant plant and a wild-type plant resulted in the production of a plant with a nuclear DNA content consistent with that plant being a tetraploid. We performed a genome wide estimation of heterozygosity on this plant and found that all chromosomes had an extreme reduction in heterozygosity. To identify the parental origin of the chromosomes of this plant, we selected 18 genomic regions in which there were polymorphisms between the two parental lines. Targeted sequencing of these regions demonstrated that all nine chromosomes were inherited from the *CENH3* wild-type parent. Taken together, these data support a model in which whole genome elimination of the parental genome from the *cenh3* mutant line occurred during the genesis of line CM21. Following genome elimination, we hypothesize that two rounds of chromosome doubling occurred shortly thereafter, resulting in the observed DNA content for line CM21, which is consistent with that line being a tetraploid.

In our analysis of the progeny produced by *cenh3* mutant plants we also observed a plant that appears to be aneuploid. This line, CM23, appears to possess a single copy of chromosome 7. Since this copy of chromosome 7 contained the wild-type *CENH3* sequence, it is likely that the maternal copy of chromosome 7 was lost following fertilization. Notably, the *cenh3* mutant line that gave rise to a progeny that only inherited one parental genome, CM21, and the line that gave rise to an aneuploid progeny, CM23, both have the same *cenh3* genotype. These lines contain two alleles of *cenh3*: one allele encodes a CENH3 protein with the E58K amino acid substitution, and the other allele encodes a protein with E58K and R60H substitutions.

Our analysis cannot determine which of these two alleles is responsible for the genome modification effects that we observed in the progeny, or if the effects are caused by interaction between these two alleles. We did not observe any progeny with evidence of chromosome loss in crosses we performed with *cenh3* mutant lines homozygous for the E58K allele. Therefore, it may be that the combination of the E58K and R60H mutations was responsible for the effects we observed. Future work will be needed to understand the contribution of each of these alleles to genome alteration.

In Arabidopsis, haploid induction rates up to 44% have been reported for plants expressing CENH3 variants with single amino acid substitutions (Kuppu et al., 2015; Kuppu et al., 2020). Because we only observed a single progeny plant in our study that demonstrated evidence of genome elimination it is not possible to accurately estimate the frequency with which genome elimination might be occurring in carrot plants carrying the *CENH3* mutations used in our study. Future work in which more progeny are screened from plants with the same genotype as the cenh3 mutant line that produced this candidate genome elimination event will be needed to determine the rate of genome elimination in these lines.

We also observed that the germination rate of the seed harvested from our crosses between the *cenh3* mutant lines and wild-type plants varied widely, ranging from 0.7% to 84.5%. For example, three crosses resulted in extremely low germination rates, ranging between 0.7% to 4.1%. All three of these crosses involved a *cenh3* mutant parent with two *cenh3* alleles. One allele encodes a CENH3 protein with the E58K mutation, and the other allele encodes a protein with R57H, E58K, and R60H mutations. Further testing will be needed to determine the role, if any, of the *cenh3* mutations on the observed low germination rates.

In addition to low germination rates, a large percentage of the viable progeny were the result of self-pollination of the *cenh3* mutant parent. The wild and cultivated forms of *D. carota* are andromonoecious and protandrous, and therefore are highly outcrossing (Linke et al., 2019). For this reason, it would be expected that there would be a larger percentage of F_1_ progeny produced. The absence of a greater number of F_1_ progeny could be explained by non-viability of the F_1_ progeny. If haploid induction or chromosome elimination occurred when a *cenh3* mutant plant was pollinated by a wild-type plant, it is possible that the progeny were not viable due to uncovering of deleterious recessive alleles or due to chromosomal imbalances caused by aneuploidy. In *Arabidopsis*, mutations in *CENH3* that lead to high haploid induction often display high rates of seed death in the progeny (Kuppu et al., 2015; Maheshwari et al., 2015; Kuppu et al., 2020). The correlation between high rates of haploid induction and seed death is hypothesized to be due to the presence of aneuploidy or imbalances between the male- and female-derived genomes in the endosperm, which can affect endosperm development (Kuppu et al., 2020). For example, in the cross between the *cenh3* mutant line RP31 and the inbred line W255-02 that we performed for this study, the germination rate of the seedlings harvested from RP31 was 15%. In comparison, the seedlings harvested from W255-02 had a 79% germination rate. The lower germination rate of the seedlings where the *cenh3* mutant parent was the female could be explained by failure of endosperm development, which usually leads to seed abortion (Birchler, 1993).

It is also possible that the crosses between *cenh3* mutant plants and wild-type plants resulted in a greater number of aneuploid plants than we detected through our screening. In other studies that crossed *cenh3* mutant plants with wild-type plants, aneuploid progeny were common (Ravi and Chan, 2010; Karimi-Ashtiyani et al., 2015; Kuppu et al., 2015; Maheshwari et al., 2015; Kelliher et al., 2016; Kuppu et al., 2020; Lv et al., 2020; Marimuthu et al., 2021; Wang et al., 2021). Our method for initially screening plants for potential genome elimination would only have identified plants that lost the region of chromosome 7 encoding CENH3 from the *cenh3* mutant plant. Therefore, aneuploid plants that retained this segment of chromosome 7 from the *cenh3* mutant plant, but lost other chromosomes, would not have been identified. For this reason, our analysis has likely underestimated the number of aneuploid progeny produced by these crosses. Future work will screen more thoroughly for aneuploid plants missing other chromosomes.

This study provides an example of how modifications of *CENH3* in carrot can result in chromosome-level changes in the progeny. In one instance, we observed evidence of elimination of all the chromosomes derived from the parent with mutant copies of *CENH3*. This outcome is consistent with the haploid inducer function of *cenh3* mutant lines previously described in Arabidopsis, maize, and wheat (Ravi and Chan, 2010; Karimi-Ashtiyani et al., 2015; Kuppu et al., 2015; Maheshwari et al., 2015; Kelliher et al., 2016; Kuppu et al., 2020; Lv et al., 2020; Marimuthu et al., 2021; Wang et al., 2021). The unexpected twist with our experiment is that the carrot plant that appears to have undergone genome elimination ended up with four identical copies of each chromosome. We hypothesize that this plant was produced by haploid induction, but then underwent two rounds of spontaneous genome doubling early in its genesis. Spontaneous chromosome doubling after the induction of haploids has been previously reported in maize and is likely genotype dependent (Ren et al., 2017). Unfortunately, this putative tetraploid plant died for unknown reasons before it reached the reproductive stage, so we were not able to evaluate the ploidy of the gametes produced by this plant. The ploidy of the germline of this plant is therefore unknown. We did not observe any evidence of higher ploidy in leaf tissue from the control plants that we analyzed by flow cytometry. Future work will be needed to determine if this type of putative quadrupled haploid is a common outcome in progeny of *cenh3* mutant plants in carrot, or if it is a rare anomaly. Regardless, however, our work provides evidence that modifying *CENH3* in carrot can lead to genome elimination and chromosome abnormalities, both of which are outcomes consistent with these mutant lines having the potential to serve as haploid inducers for future use in plant breeding.

## Data availability statement

The original contributions presented in the study are publicly available. The DNA sequence data described in this paper can be found here: https://www.ncbi.nlm.nih.gov/sra/PRJNA1034105. Accession number: PRJNA1034105.

## Author contributions

CM: Conceptualization, Formal Analysis, Investigation, Methodology, Writing – review & editing, Data curation, Visualization, Writing – original draft. IG: Conceptualization, Formal Analysis, Investigation, Methodology, Writing – review & editing, Funding acquisition, Project administration, Supervision. PK: Conceptualization, Formal Analysis, Funding acquisition, Investigation, Methodology, Project administration, Supervision, Writing – review & editing.
