# Supplementary material for: Chromosome-level changes and genome elimination by manipulation of CENH3 in carrot (Daucus carota)
Source: Front Plant Sci. 2023 Nov 15;14:1294551. doi: 10.3389/fpls.2023.1294551 (PMC10684906; doi:10.3389/fpls.2023.1294551)
Supplement: Supplementary file 1 [file Table_1.docx]

Supplementary Tables

**Supplementary Table S1.** Regions of the mitochondrial genome were PCR amplified using the primers NAD7-F1 and NAD7-R1 (see materials and methods). The DNA sequence of these PCR products was then determined by Sanger sequencing using primer NAD7-R1 as the sequencing primer. This table reports the DNA sequences obtained for the two parental lines used in the genetic cross that produced progeny plant CM21 (RP31 and W255-02), and the DNA sequence of the CM21 plant.

| Sample | Sequence |
| --- | --- |
| RP31 | TGAAATAGGAGGCCGGGTTCTCTTTCTACGACCCTTTGTTTTTTATATGATAGGCACGGACTCCGCGAACGTCCCGCGCCCTTTACT**AATACT**AATAAGGGAAAGAAAGCCTCAACCAGAACCACATTCCTTTTGCG**T**GCGGGTGTAGCTAAGTGTCTTACTCTATTGGTCATAGTTTCCTGCTGTTGCGGCCGGTGCT**C**GTTTGCGCGCGCGTGAACCAACCCAACAAACAAGGAAAGGACGCCTCTCTGGGCATCTGAGAATGATTCGAGCCGTATGAAGGGAAACTCTCACGTACAGTTTTTTTG**G**GGGGGGCCGGCAGGGTCCCCCCACTTACTTGGCCCGGGCCT |
| W255-02 | TGAAATAGGAGGCCGGGTTCTCTTTCTACGACCCTTTGTTTTTTATATGATAGGCACGGACTCCGCGAACGTCCCGCGCCCTTTACT**------**AATAAGGGAAAGAAAGCCTCAACCAGAACCACATTCCTTTTGCG**G**GCGGGTGTAGCTAAGTGTCTTACTCTATTGGTCATAGTTTCCTGCTGTTGCGGCCGGTGCTTGTTTGCGCGCGCGTGAACCAACCCAACAAACAAGGAAAGGACGCCTCTCTGGGCATCTGAGAATGATTCGAGCCGTATGAAGGGAAACTCTCACGTACAGTTTTTTTG*-*GGGGGGCCGGCAGGGTCCCCCCACTTACTTGGCCCGGGCCTAAGTGAA |
| CM21 | TGAAATAGGAGGCCGGGTTCTCTTTCTACGACCCTTTGTTTTTTATATGATAGGCACGGACTCCGCGAACGTCCCGCGCCCTTTACT**AATACT**AATAAGGGAAAGAAAGCCTCAACCAGAACCACATTCCTTTTGCG**T**GCGGGTGTAGCTAAGTGTCTTACTCTATTGGTCATAGTTTCCTGCTGTTGCGGCCGGTGCT**C**GTTTGCGCGCGCGTGAACCAACCCAACAAACAAGGAAAGGACGCCTCTCTGGGCATCTGAGAATGATTCGAGCCGTATGAAGGGAAACTCTCACGTACAGTTTTTTTG**G**GGGGGGCCGGCAGGGTCCCCCCACTTACTTGGCCCGGGCCT |

**Supplementary Table S2.** Positions and genomic sequences of regions of the nuclear genome used for identification of the parental origin of the chromosomes of CM21. The sequences indicated below correspond to the carrot reference genome assembly ASM162521v1. “Position 1” and “Position 2” indicate the coordinates of the sequenced region on the reference genome.

| Name | Position 1 | Position 2 | Chromosome | Genomic sequence |
| --- | --- | --- | --- | --- |
| C1-A | 1208308 | 1208750 | 1 | CCATGCGAATAGAAGTTAAGGCATATATATTATTATCAAGACCTCGTACTTAATCTTCGTAGTACGCTATTTAATTGGTATCGTAGTGACAAGTTTAAGTTGTAACTGACTAGCAGTCTAGCATTAATCAATTCAGAGGCTTGGTGTGTGTTTGTGCCGATATTCTCTATATGTTTGATTAGTAAAAAGAGATGATTTATTATTCTGATGAATCATTAGACAGACTCTCAGTATCTACAAGCTTAATGATTGATATTTGTAGTTTAGGCCCTAAAGCTTTTAAATAATTTTTGCTGATCCTTTTGTTGCTCTGTATACAACAATAAACTAATCATGGCTTCATGGTGTTAGGATTTGGCTTTCGATTTGTCTCTATCAGCATTGCTGGGTGAAAACATTTACAATTTCGGTGAATTGCTTGCACAT |
| C2-A | 6716586 | 6717033 | 2 | AAACGAATGTATGGCGACCTGCCTTATCGTCTGGTAAGTATAGTTAATGTACTAGCTTATCTTTATTCCTACTATTTATAATTGGTCTCTTAAGACTTATTATGTAAACAATTTGATGAAAAGTGATGAATAAAACTCATTTGTTTAAATGAGTTACAACATTGATGAATGCGACTTAACTGACAATGGAAGATGCACTCAGTATAAGTTAATACAGACAATTAATCTGTAAGTAGAAGTGTACATTATATGGTAATTGAAAATTAGGAGATAACACTGTTCTTGTCAGTTTAAATACTCTAAGGAGCCTATTTTGTCCATCTGAAGTCTCCAAACTTTTTCTCTAATTTGTTAATTTTATATTCCAATAGCATGAATGCTTCTGCGAGTGGATTTCCATGTACAAGCTACCTAAGGAGAGTGAAATTGTGAAGCTAAGCCCGAGAGT |
| C3-A | 25137622 | 25137847 | 3 | AGCTCTTGACCAGTCTTCCTACAACTATTGTTGATCTTGAGCCTCATCAGATCCATACGTTTTATGAATCTGTTAGTTACTATAACTTTAATATTCTCTCTGACTACATCAGGGCTCCTATGCTTGGCTGATTAAGAGAAAGTCTTATTTTAACATTTGATTCTCATGGTTTTTACTAGGTTGGTACTATGATTCAAGCAGAATCTGACCCGCAGAAGAGAGATGA |
| C4-A | 6425817 | 6426156 | 4 | TGGGTGATGTTGAGAAAGTGCTAAGGTTTAATTGGAGGGCTACATGTGTTGACATTAAGGTCGATATAGATAAGTCCTCATTGCTCGACATTATATATATTTAATTATGATGACCGTACCAAGATGAGATTGGATTTCCCACACTTTCGGTAATGTATTTAACATGGCCCATCATCCATTAGAGACTAATCAAGATTTGACAAGAATGTTTGAGAGGTTTAGTTATACGGAGCTAATTGACAATTAGGGGGTAATGTTATAAAACCAGCTAGCATTTGTAAATTGGTTTTCAATTTTAGGTGATCACATGAAGAACATGAGACGGTGGATAATGGTGAGC |
| C5-A | 2162758 | 215983 | 5 | AAATTGGTGAAGCTCCTGTTGTAACCGAAGGCAAATGAATGCTTGAAAAATCAGTTTCAGCTGTTTCGGGGATGCCCTAAAAGTAGGTTTAATACTTTCGTGATTACGATAAATAGGCTAAAACACATGCACACACAGTAAACTAAAAATAAGCAACACAAAAATGGACAAGCATACAGAATACAGATAGTCCACAGCTCTCTCTGGATTGTTATAAGCAGCTCGA |
| C6-A | 2469849 | 2470250 | 6 | AAGCCTTGTATCCACACTTAAGACTTCTAAAATGGGAAGCTTTTGTTTTTAAATGCAATTAATTAAACCCTCCAATTTAATACATTAGGTTAGCCTCGTAGTCCTCTGTTTAATATTCCTCTCTTCCTTTTCCACATGAATTAAGTACTTTGATACAGGAAATATAAGGGAATCAAGCCTTGCATAAGCATGTATGATGTACTTGTTCATTTGGGTTTTTGGATTTTATAAGATAGTGGTTTCAGAAAGAGCATATATTAACAAATACTGATGCCTATTATACATCCCCGCAGGTACAATAATATCTTTATCCTTATCATTGGTGCCATTGTGTTCATATGCGCGACTGTCGGTGTGTTGCTAGTAATGGAAACACTAAGTGCCTTTTTGCATGCCTTGCGT |
| C7-A | 5006688 | 5006903 | 7 | CTCTTCCTTCCCCATTGTGTGGTTACAAGTTTTGCTTTCTTGGTGTTCTGATTAGTGCTTTACTTGGTAAACTAATCCCATATAAATTTTTTTGTGCCAGTTTCAGATTATTCTGGGATCCATGTCAAGTGCCTACTCATTAACCAAAAAGATTGAATGCTTTGTGCCAGGTTATAGTTGCAGAA |
| C8-A | 1674123 | 1674345 | 8 | GGTAATACGTGATCTGCCCTCAGTTATTCTCACATTACATAGGAGTATTTACTTTTATCTCAATAATTTTTTTGGAAAGATCACTAGTAGAGGTAATAAGGTCTGATCACATTTATATTTGCTTCAAAATACGATATTTGGAAGATTAAGTGATTAGTAAATTTGTATATTCCAGGAAGTTCAAGTGCAAAAAGACTGATCCCCGGGATTGTTGGTGGTTTAT |
| C9-A | 1426000 | 1426335 | 9 | AACTAAATGAGAACAAATGTTGAACACATGTTTTAAATACAACAAGACAACATCATCGTTTGAGTATCATATATAAGATATCTGTCGAATTCACAACTTCTTATGCAGTCATGCTATATCTCTTGTGGATGCCCCATGACTTCCATATATCCCCGTAAAGGGAGCCCAGAACTTAACTGTTTCACATTAGGATATAACAATACATATTTCGCCAGAATTCGTGGCAAATTTGATGCTGGCAAGTACTTGCACAACTTCAAGCATGCTGGTATCTTATTGTCTCATTAGTGAACTGAATATATTACTGGATAACGATGTTGGGCAGTGATGCAGTTT |
| C1-B | 36730905 | 36731123 | 1 | GGATATCCATTTCAGCAAGCACATTACTGTTAATTGCTAGTGCTGCCTTCAGTATCTGGTTGGTGCAGTCCCTGCACTGCTGCAGCTTCTTCCTAGCATCCTCCGTCAACCCATTTGGTGGAACCTTAGGACAAGGTAGCCACCACTTCTCTTCCTGCCTAACTGACGGCCTCCCACTTGAAAAAGCAGATGGATAGGTATCACAATCATCTCCAGCCACACCACGTTCAACATACCAAAATTCCAAATC |
| C2-B | 34585650 | 34585921 | 2 | CACATGATGGGTACTCGGTAAGAGTTCTATATATATCTTGAGGTATCCTGACAAATTATCTATCACAGTGATAATGACATTCAATGTGAAATCATGATAGATTATATGAATATATCATGAGCAAGTGATAAGCTATATCCTACTGACACTAAACGAATTAAAATTTGATTCTATTCTTTTTCAGAACGAATACTGTATGATGTATTTTATTCTGTTATTCAGAGCCATTCTTCTTCTTTGACTGACGATGAAGTAAAGGATGCCCGTTCTGGTAAAAATGAGGTTGAAAAAACTATTCGTGCAGTTTATGCACAAAAAAAGG |
| C3-B | 5583779 | 5584018 | 3 | GTTGAGTTAAGTTGTTGACGGAAAATGGGATTTTTCCGGTGAAGGCGTTGGAAGAGAGATCCAAACGGACTAACCGAGTTAGACTGAGTAGACTCGGTGGGAACTCGGAGCTGAACTCGTTGTTTTGGAGGTAAAGGCTCCGCAGGAGCTTCAGGTTCGAGAAATCCGGTGGGAGTGAACCGGTGAGTCCATTGGACCGGAGACTCAGAACTCGGAGCTGAGTCAAGTTACCGAGTGTGTTCGGTGGGATTGAGCCAACTAAGCCAACACCGGGTAGCCGTAGAG |
| C4-B | 32757411 | 32757729 | 4 | GTCCAGCCACAAAGACGAACACCACTCAACACTACTCTGCTGAAGAAACAAAAGAATGCGGTGGGGAGGGCTGGCCAGTGTTTTCACCATTGAGAGGATTTCCTCAGGAGTTCTCTCTGGTTCACCCTTCTTTGCCTGACAAATGCCAATTCAATTCATATCAGTGAAAATTACAGATTTCATAGGGTAGCAGAAATAAGTATAATCACATTTTCAGATTGATTACCTTAAGAACAAATCCTGGGGTATCAATGATTGTGAGATTAGGACAATGGGCATATTCAGCTCTCATTACAATTGGTTTAGAAGAGACAGCAGC |
| C5-B | 36840460 | 36840761 | 5 | AGAGTGGATCACATTCGCCCATCTTCCAAGCCATAACTTTGTTTTCATGTACATGATAAATTATGAGTTGTGATACTATTTTTAAGCGACAAGTTGACTTCAAACCCTAGGGAAAAACTTTTTTTACCTGGATCATCGTATTCTAACAAATCAAATGATAATTATAACAAAATTGTGTGCAGGAAAATCACCAGTAACAATTACCAATTTATTCTTCGCGAAAGAGCAATCTGTAAGGACTGCTTCAAGAAGCATAATTCTCAGAGCCTCATAATTTCTTGGAAATCAAGATCATACCAGGCTGATCGTTGTTATGAAGCTACAAAGGATGAGAGAGCATACGAGGGAGATGATAGAGACATTCAAGATCGAGTACA |
| C6-B | 30825901 | 30826166 | 6 | GGTTTCAACAATTCCACAATCACATTATGATTGTAAGAGCAACAAACTGTGTTACCACTATCACAACAAAATACATTGTCGTGTTATGGTAGACAAGTTATAGTTAATCTGTAATGAAAGCAAAACTTAAAGGCCACCGGAATAGATAAGTTATTGGCAGCTCAAAGATTTATTCACCTTACGTTCACTGCTTTGTACTGTAAAAGGTCTAGTCTTGACATCAAGGAATTCAACATCTTCTTGAAACAATTCTGCACCCCAACGTT |
| C7-B | 28263985 | 28264194 | 7 | ATGACCACGTCCACGTAACTTGCCTTGTTATCTAAAATTCTCCTACTACCAAACCTGGCCATTGGCCAACTGGTTCATTATATGCTGCTGATTTTCTTGACTAATCTTATATGCGTCCACATTCATGAACCGGTGGGTTTGATTTCAAACGTGAAATTTATTACGTACGAATAATACTATTTCGATTTAACGAGACAGAGAATTCAAAAGCAAGTGGTCATTAGTTCAAATCACATGCTTAAGAAAAAACAAAAATGCATCTAA |
| C8-B | 26612288 | 26612490 | 8 | AGCTGCAAAGTCTTCCTCTGGACATTTGTTTGCTGCGTATGGTTCCCTAATAAAGCCATCATTTGAAAAGATCCTAGTGCAGCCTGGTGAGGACATTAAGTTGAATAGTTCCGCAGATGGAATTCTCTTACCACATGGTCAGATCAAGAAAGGCAAGAAAGGGTCGACTGCGCTTACCCAAAGTAGGTTCCTCACTCTTGTTGAAAAGTCATTTGATCATGAT |
| C9-B | 25860649 | 25860908 | 9 | CCAGAGAGAGCGAAGAAAACCCGCATTAACACCATCCGGGCCCGGCGCTTTAGTGCCATCTGTGGCGGCCAGGGCATCTTCAACCTCTTGTAGCGAGAAAGCCTTGATCAGGTCATATTTTTCCTCAGGTGATAACGAAGGTAGTAGACCCTTCCCCAGGAGGAATATATTGTCCGAAAGAGGTCGAGTAAGTAGAGATTTAAAATGGTCAAAAAGGAAGTTTTTGATCTCTATAGGTTTGAAGATCAAATTACCGTCCTTCACCAGAGAAATAATATTGTTCGAGTGGTTCCT |

**Supplementary Table S3.** PCR primers used to amplify regions of the carrot nuclear genome for Sanger sequencing. These PCR primers were used to amplify the DNA sequence regions indicated in Supp Table S3.

| Primer name | Sequence | Fragment size (bp) |
| --- | --- | --- |
| C1-A-F1 | CCATGCGAATAGAAGTTAAGGCA | 426 |
| C1-A-R1 | ATGTGCAAGCAATTCACCGA |  |
| C2-A-F1 | AAACGAATGTATGGCGACCTG | 448 |
| C2-A-R1 | ACTCTCGGGCTTAGCTTCAC |  |
| C3-A-F1 | AGCTCTTGACCAGTCTTCCT | 220 |
| C3-A-R1 | TCATCTCTCTTCTGCGGGTC |  |
| C4-A-F1 | TGGGTGATGTTGAGAAAGTGC | 226 |
| C4-A-R1 | GCTCACCATTATCCACCGTC |  |
| C5-A-F1 | CCATTTGTTTAGGTTCCAGCTCA | 250 |
| C5-A-R1 | GGAATGGCTGCTGGTAGTTAC |  |
| C6-A-F1 | GGTTAGCCTCGTAGTCCTCTG | 227 |
| C6-A-R1 | CTAGCAACACACCGACAGTC |  |
| C7-A-F1 | CTCTTCCTTCCCCATTGTGTG | 185 |
| C7-A-R1 | TTCTGCAACTATAACCTGGCAC |  |
| C8-A-F1 | GGTAATACGTGATCTGCCCTC | 223 |
| C8-A-R1 | ATAAACCACCAACAATCCCGG |  |
| C9-A-F1 | CAACAAGACAACATCATCGTTTG | 285 |
| C9-A-R1 | TGCCCAACATCGTTATCCAG |  |
| C1-B-F1 | ATCCATTTCAGCAAGCACATTAC | 219 |
| C1-B-R1 | GGTGTGGCTGGAGATGATTG |  |
| C2-B-F1 | CACATGATGGGTACTCGGTAAG | 272 |
| C2-B-R1 | CCAGAACGGGCATCCTTTAC |  |
| C3-B-F1 | TTTTCCGGTGAAGGCGTTG | 285 |
| C3-B-R1 | GGTGTTGGCTTAGTTGGCTC |  |
| C4-B-F1 | TGACAAAGAAGGAGGTGGCA | 266 |
| C4-B-R1 | TCTTTGCCTCTGACCCTGAC |  |
| C5-B-F1 | ACATTCGCCCATCTTCCAAG | 302 |
| C5-B-R1 | ACAACGATCAGCCTGGTATG |  |
| C6-B-F1 | GGTTTCAACAATTCCACAATCAC | 266 |
| C6-B-R1 | AACGTTGGGGTGCAGAATTG |  |
| C7-B-F1 | GTCCACGTAACTTGCCTTGT | 210 |
| C7-B-R1 | ACCACTTGCTTTTGAATTCTCTG |  |
| C8-B-F1 | CTGCAAAGTCTTCCTCTGGAC | 203 |
| C8-B-R1 | TTCAACAAGAGTGAGGAACCTAC |  |
| C9-B-F1 | AAACCCGCATTAACACCATCC | 260 |
| C9-B-R1 | ATTATTTCTCTGGTGAAGGACGG |  |

**Supplementary Table S4.** DNA sequences of regions in the nuclear genome of individual lines used to identify the parental origin of the nuclear genome of CM21. DNA sequences were determined by Sanger sequencing of the PCR amplicons from individual plants. Red letters in bold indicate polymorphisms between the indicated line and the carrot reference genome.

| Primer name | Sample | Sequence |
| --- | --- | --- |
| C1-A-F1 | RP31 | TCTTCGTAGTACGCTATTTAATTGGTATCGT**A**GTGACAAGTTTAAGTTGTAACTGACTAGCAGTCTAGCATTAATCAATTCAGAGGCTTGGTGTGTGTTTGTGCCGATATTCTCTATATGTTTGATTAGTAAAAAGAGATGATTTATTATTCTGATGAATCATTAGACAGACT**C**TCAGTATCTACAAGCTTAATGATTGATATTTGTAGTTTAGGCCCTAAAGCT**T**TTAAATAATTTTTGCTGATCCTTTTGTTGCTCTGTATACAACAATAAACTAA**T**CATGGCTTCATGGTGTTAGGATTTGGCTTTCGATTTGTCTCTATCAGCATTGCTGGGTGAAAACATTTACAATTTCGGTGAATTG |
| C1-A-F1 | W255-02 | TCTTCGTAGTACGCTATTTAATTGGTATCGT**G**GTGACAAGTTTAAGTTGTAACTGACTAGCAGTCTAGCATTAATCAATTCAGAGGCTTGGTGTGTGTTTGTGCCGATATTCTCTATATGTTTGATTAGTAAAAAGAGATGATTTATTATTCTGATGAATCATTAGACAGACT**T**TCAGTATCTACAAGCTTAATGATTGATATTTGTAGTTTAGGCCCTAAAGCT**C**TTAAATAATTTTTGCTGATCCTTTTGTTGCTCTGTATACAACAATAAACTAA**C**CATGGCTTCATGGTGTTAGGATTTGGCTTTCGATTTGTCTCTATCAGCATTGCTGGGTGAAAACATTTACAATTTCGGTGAATTG |
| C1-A-F1 | CM21 | TCTTCGTAGTACGCTATTTAATTGGTATCGT**G**GTGACAAGTTTAAGTTGTAACTGACTAGCAGTCTAGCATTAATCAATTCAGAGGCTTGGTGTGTGTTTGTGCCGATATTCTCTATATGTTTGATTAGTAAAAAGAGATGATTTATTATTCTGATGAATCATTAGACAGACT**T**TCAGTATCTACAAGCTTAATGATTGATATTTGTAGTTTAGGCCCTAAAGCT**C**TTAAATAATTTTTGCTGATCCTTTTGTTGCTCTGTATACAACAATAAACTAA**C**CATGGCTTCATGGTGTTAGGATTTGGCTTTCGATTTGTCTCTATCAGCATTGCTGGGTGAAAACATTTACAATTTCGGTGAATTG |
| C2-A-R1 | RP31 | TCCACTCGCAGAAGCATTC**A**TGCTATTGGAATATAAAATTAACAAATTAGAGAAAAAGTTTG**G**AGACTTCAGATGGACAAAATAGGCTCCTTAGAGTATTTAAACTGACAAGAACAGTGTTATCTCCTAATTTTCAATTACCATATAATGTACACTTCTACTTACAGATTAATTGTCTGTATTAACTTATACTGAGTGCATCTTCCATTGTCAGTTAAGTCGCATTCATCAATGTTGTAACTCATTTAAACAAATGAGTTTTATTCATCACTTTTCATCAAATTGTTTACATAATAAGTCTTAAGAGACCAATTATAAATAGTA**G**GAATAAAGATAAGCTAGTACATTAACTATACTTACCAGACGATAAGGCAGGTCG |
| C2-A-R1 | W255-02 | TCCACTCGCAGAAGCATTC**G**TGCTATTGGAATATAAAATTAACAAATTAGAGAAAAAGTTTG**[G/C]**AGACTTCAGATGGACAAAATAGGCTCCTTAGAGTATTTAAACTGACAAGAACAGTGTTATCTCCTAATTTTCAATTACCATATAATGTACACTTCTACTTACAGATTAATTGTCTGTATTAACTTATACTGAGTGCATCTTCCATTGTCAGTTAAGTCGCATTCATCAATGTTGTAACTCATTTAAACAAATGAGTTTTATTCATCACTTTTCATCAAATTGTTTACATAATAAGTCTTAAGAGACCAATTATAAATAGTA**[T/G]** GAATAAAGATAAGCTAGTACATTAACTATACTTACCAGACGATAAGGCAGGTCG |
| C2-A-R1 | CM21 | TCCACTCGCAGAAGCATTC**G**TGCTATTGGAATATAAAATTAACAAATTAGAGAAAAAGTTTG**C**AGACTTCAGATGGACAAAATAGGCTCCTTAGAGTATTTAAACTGACAAGAACAGTGTTATCTCCTAATTTTCAATTACCATATAATGTACACTTCTACTTACAGATTAATTGTCTGTATTAACTTATACTGAGTGCATCTTCCATTGTCAGTTAAGTCGCATTCATCAATGTTGTAACTCATTTAAACAAATGAGTTTTATTCATCACTTTTCATCAAATTGTTTACATAATAAGTCTTAAGAGACCAATTATAAATAGTA**T**GAATAAAGATAAGCTAGTACATTAACTATACTTACCAGACGATAAGGCAGGTCG |
| C3-A-R1 | RP31 | AAAACCATGAGAATCAAATGTTAAAATAAGACTTTCTCTT**A**ATCAGCCAAGCATAG**G**AGCCCTGATGTAGTCAGAGAGAATATTAAAGTTATAGTAACTAACAGATTCATAAAACGTATGGATCTGATGAGGCTCAAGATCAACAATAGTTGTAGGAAGACTGGTCAAGA |
| C3-A-R1 | W255-02 | AACCATGAGAATCAAATGTTAAAATAAGACTTTCTCTT**G**ATCAGCCAAGCATAG**T**AGCCCTGATGTAGTCAGAGAGAATATTAAAGTTATAGTAACTAACAGATTCATAAAACGTATGGATCTGATGAGGCTCAAGATCAACAATAGTTGTAGGAAGACTGGTCAAGA |
| C3-A-R1 | CM21 | AACCATGAGAATCAAATGTTAAAATAAGACTTTCTCTT**G**ATCAGCCAAGCATAG**T**AGCCCTGATGTAGTCAGAGAGAATATTAAAGTTATAGTAACTAACAGATTCATAAAACGTATGGATCTGATGAGGCTCAAGATCAACAATAGTTGTAGGAAGACTGGTCAAGAG |
| C4-A-R1 | RP31 | CAATTTACAAATGCTAGCTGGTTTTATAACATTACCCCCTAATTGTCAATTAGCTCCGTATAA**C**TAAACCTCTCAAACATTCTTGTCAAATCTTGATTAGTCTCTAATGGATGATGGGCCATGTTAAATACATTACCGAAAGTGTGGGAAATCCAATCTCATCTTGGTACGGTCATCATAATTAAATATATATAAT**G**TCGAGCAATGAGGACTTATCTATATCGACCTTAATGTCAACACATGTAGCCCTCCAATTAAACCTTAGCACTTTCTCAACATCACC |
| C4-A-R1 | W255-02 | CAATTTACAAATGCTAGCTGGTTTTATAACATTACCCCCTAATTGTCAATTAGCTCCGTATAA**A**TAAACCTCTCAAACATTCTTGTCAAATCTTGATTAGTCTCTAATGGATGATGGGCCATGTTAAATACATTACCGAAAGTGTGGGAAATCCAATCTCATCTTGGTACGGTCATCATAATTAAATATATATAAT**A**TCGAGCAATGAGGACTTATCTATATCGACCTTAATGTCAACACATGTAGCCCTCCAATTAAACCTTAGCACTTTCTCAACATCACC |
| C4-A-R1 | CM21 | CAATTTACAAATGCTAGCTGGTTTTATAACATTACCCCCTAATTGTCAATTAGCTCCGTATAA**A**TAAACCTCTCAAACATTCTTGTCAAATCTTGATTAGTCTCTAATGGATGATGGGCCATGTTAAATACATTACCGAAAGTGTGGGAAATCCAATCTCATCTTGGTACGGTCATCATAATTAAATATATATAAT**A**TCGAGCAATGAGGACTTATCTATATCGACCTTAATGTCAACACATGTAGCCCTCCAATTAAACCTTAGCACTTTCTCAACATCACC |
| C5-A-R1 | RP31 | TCTGGGGTCCTTTGTACTTGTACCAACTGGAAGTAACAAGAAAAACAGCTGCATTGAGGAC**A**GAAAGTGCAGCCAAGAGGTA**A**TAGAAATATTCCAGGCTGCCCTTGTTAAGATCTTCTGCCAACCAGTCTCCACCAGC**A**GTCTGTTTTGTGGTCATGTGAACCGTTCCGATGAGAAA**G**CCATAGAAGTAACTACCAGCAGCCATTC |
| C5-A-R1 | W255-02 | TCTGGGGTCCTTTGTACTTGTACCAACTGGAAGTAACAAGAAAAACAGCTGCATTGAGGAC**T**GAAAGTGCAGCCAAGAGGTA**G**TAGAAATATTCCAGGCTGCCCTTGTTAAGATCTTCTGCCAACCAGTCTCCACCAGC**C**GTCTGTTTTGTGGTCATGTGAACCGTTCCGATGAGAAA**A**CCATAGAAGTAACTACCAGCAGCCATTC |
| C5-A-R1 | CM21 | TCTGGGGTCCTTTGTACTTGTACCAACTGGAAGTAACAAGAAAAACAGCTGCATTGAGGAC**T**GAAAGTGCAGCCAAGAGGTA**G**TAGAAATATTCCAGGCTGCCCTTGTTAAGATCTTCTGCCAACCAGTCTCCACCAGC**C**GTCTGTTTTGTGGTCATGTGAACCGTTCCGATGAGAAA**A**CCATAGAAGTAACTACCAGCAGCCATTC |
| C6-A-F1 | RP31 | GTACTTT**G**AT**A**CAGGAAATATAAGGGAATCAAGCCTTGCATAAGCATGTATGATGTACTTGTTCATTTGGGTTTTTGGATTTTATAAGATAGTGGTTTCAGAAAGAGCATATATTAACAAATACTGATGC**C**TATTATACATCCCCGCAGGTACAATAATATCTTTATCCTTATCATTGGTGCCATTGTGTTCATATGCGCGACTGTCGGTGTGTTGC |
| C6-A-F1 | W255-02 | GTACTTT**[G/A]**AT**T**CAGGAAATATAAGGGAATCAAGCCTTGCATAAGCATGTATGATGTACTTGTTCATTTGGGTTTTTGGATTTTATAAGATAGTGGTTTCAGAAAGAGCATATATTAACAAATACTGATGC**[C/T]**TATTATACATCCCCGCAGGTACAATAATATCTTTATCCTTATCATTGGTGCCATTGTGTTCATATGCGCGACTGTCGGTGTGTTGC |
| C6-A-F1 | CM21 | GTACTTT**A**AT**T**CAGGAAATATAAGGGAATCAAGCCTTGCATAAGCATGTATGATGTACTTGTTCATTTGGGTTTTTGGATTTTATAAGATAGTGGTTTCAGAAAGAGCATATATTAACAAATACTGATGC**T**TATTATACATCCCCGCAGGTACAATAATATCTTTATCCTTATCATTGGTGCCATTGTGTTCATATGCGCGACTGTCGGTGTGTTGC |
| C7-A-F1 | RP31 | TTACTTGGTAAACTAATCCCA**T**ATAAATTTTTTTGTGCCAGTTTCAGATTATTCTGGGATCCATGTCAAGTGCCTACTCATTAACC**A**AAAAGATTGAATGCTTTGTGCCAGGTTATAGTTGCAGAAA |
| C7-A-F1 | W255-02 | TTACTTGGTAAACTAATCCCA**C**ATAAATTTTTTTGTGCCAGTTTCAGATTATTCTGGGATCCATGTCAAGTGCCTACTCATTAACC**G**AAAAGATTGAATGCTTTGTGCCAGGTTATAGTTGCAGAAA |
| C7-A-F1 | CM21 | TTACTTGGTAAACTAATCCCA**C**ATAAATTTTTTTGTGCCAGTTTCAGATTATTCTGGGATCCATGTCAAGTGCCTACTCATTAACC**G**AAAAGATTGAATGCTTTGTGCCAGGTTATAGTTGCAGAAA |
| C8-A-F1 | RP31 | ATTTACTTTTATCTCAATAA**T**TTTTTTGGAAAGATCACTAGTAGAGGTAATAAGGTCTGATCACATTTATATTTGCTTCAAAATACGATATTTGGAAGATTAAGTGATTAGTAAATTTGTATATTCCAGGAAGTTCAAGTGC**A**AAAAGACTGATCCCCGGGATTGTTGGTGGTTTATA |
| C8-A-F1 | W255-02 | ATTTACTTTTATCTCAATAA**[T/A]**TTTTTTGGAAAGATCACTAGTAGAGGTAATAAGGTCTGATCACATTTATATTTGCTTCAAAATACGATATTTGGAAGATTAAGTGATTAGTAAATTTGTATATTCCAGGAAGTTCAAGTGC**[G/A]**AAAAGACTGATCCCCGGGATTGTTGGTGGTTTATA |
| C8-A-F1 | CM21 | ATTTACTTTTATCTCAATAA**A**TTTTTTGGAAAGATCACTAGTAGAGGTAATAAGGTCTGATCACATTTATATTTGCTTCAAAATACGATATTTGGAAGATTAAGTGATTAGTAAATTTGTATATTCCAGGAAGTTCAAGTGC**G**AAAAGACTAATCCCCGGGATTGTTGGTGGTTTATA |
| C9-A-R1 | RP31 | GCATGCTTGAAGTTGTGCAAGTACTTGCCAGCATCAAATTTGCCACGAATTCTGGC**G**AAATATGTATTGTTATATCCTAATGTGAAACA**G**TTAAGTTCTGGGCTCCCTTTACGGGGATATATGGAAGTCATGGGGCATCCACAAGAGATATAGCATGACTGC**A**TAAGAAGTTGTGAATTCGACAGATATCTTATATATGATACTCAAACGATGATGTTGTCTTGTTGA |
| C9-A-R1 | W255-02 | GCATGCTTGAAGTTGTGCAAGTACTTGCCAGCATCAAATTTGCCACGAATTCTGGC**A**AAATATGTATTGTTATATCCTAATGTGAAACA**C**TTAAGTTCTGGGCTCCCTTTACGGGGATATATGGAAGTCATGGGGCATCCACAAGAGATATAGCATGACTGC**G**TAAGAAGTTGTGAATTCGACAGATATCTTATATATGATACTCAAACGATGATGTTGTCTTGTTGA |
| C9-A-R1 | CM21 | GCATGCTTGAAGTTGTGCAAGTACTTGCCAGCATCAAATTTGCCACGAATTCTGGC**A**AAATATGTATTGTTATATCCTAATGTGAAACA**C**TTAAGTTCTGGGCTCCCTTTACGGGGATATATGGAAGTCATGGGGCATCCACAAGAGATATAGCATGACTGC**G**TAAGAAGTTGTGAATTCGACAGATATCTTATATATGATACTCAAACGATGATGNTGTCTTGTTGA |
| C1-B-R1 | RP31 | GGCCGTCAGTTAGGCAGGAAGAGAAGTGGTGGCTACCTTGTCCTAA**G**GTTCCAC**C**AAATGGGTT**G**ACGGAGGATGCTAGGAAGAAGCTGCAGCAGTGCAGGGACTGCACCAACCAGATACTGAAGGCAGCACTAGCAATTAACAGTAATGTGCTTGCTGAAATGGATAN |
| C1-B-R1 | W255-02 | GGCCGTCAGTTAGGCAGGAAGAGAAGTGGTGGCTACCTTGTCCTAA**A**GTTCCAC**G**AAATGGGTT**A**ACGGAGGATGCTAGGAAGAAGCTGCAGCAGTGCAGGGACTGCACCAACCAGATACTGAAGGCAGCACTAGCAATTAACAGTAATGTGCTTGCTGAAATGGAT |
| C1-B-R1 | CM21 | GGCCGTCAGTTAGGCAGGAAGAGAAGTGGTGGCTACCTTGTCCTAA**A**GTTCCAC**G**AAATGGGTT**A**ACGGAGGATGCTAGGAAGAAGCTGCAGCAGTGCAGGGACTGCACCAACCAGATACTGAAGGCAGCACTAGCAATTAACAGTAATGTGCTTGCTGAAATGGAT |
| C2-B-F1 | RP31 | GCTCCGAGTTCTGAGTCTCCGGTCCAATGGACTCACCGGTTCACTCCCACC**G**GATTTCTCGAACCT[**T/G**]AAGCTCCT**G**CG**G**AGCCTTTACCTCCAAAACAACGAGTTCAG**C**TCCGAGTTCCC**A**CCGAGTCTACTCAGTCTAACTCGGTTAGTCCGTTTGGATCTCTCTTCCAACGCCTTCACCGGAAAAA |
| C2-B-F1 | W255-02 | GCTCCGAGTTCTGAGTCTCCGGTCCAATGGACTCACCGGTTCACTCCCACC**T**GATTTCTCGAACCT**C**AAGCTCCT**C**CG**T**AGCCTTTACCTCCAAAACAACGAGTTCAG**T**TCCGAGTTCCC**G**CCGAGTCTACTCAGTCTAACTCGGTTAGTCCGTTTGGATCTCTCTTCCAACGCCTTCACCGGAAAAA |
| C2-B-F1 | CM21 | GCTCCGAGTTCTGAGTCTCCGGTCCAATGGACTCACCGGTTCACTCCCACC**T**GATTTCTCGAACCT**C**AAGCTCCT**C**CG**T**AGCCTTTACCTCCAAAACAACGAGTTCAG**T**TCCGAGTTCCC**G**CCGAGTCTACTCAGTCTAACTCGGTTAGTCCGTTTGGATCTCTCTTCCAACGCCTTCACCGGAAAAA |
| C3-B-R1 | RP31 | GCTCCGAGTTCTGAGTCTCCGGTCCAATGGACTCACCGGTTCACTCCCACC**G**GATTTCTCGAACCT[**T/G**]AAGCTCCT**G**CG**G**AGCCTTTACCTCCAAAACAACGAGTTCAG**C**TCCGAGTTCCC**A**CCGAGTCTACTCAGTCTAACTCGGTTAGTCCGTTTGGATCTCTCTTCCAACGCCTTCACCGGAAAAA |
| C3-B-R1 | W255-02 | GCTCCGAGTTCTGAGTCTCCGGTCCAATGGACTCACCGGTTCACTCCCACC**T**GATTTCTCGAACCT**C**AAGCTCCT**C**CG**T**AGCCTTTACCTCCAAAACAACGAGTTCAG**T**TCCGAGTTCCC**G**CCGAGTCTACTCAGTCTAACTCGGTTAGTCCGTTTGGATCTCTCTTCCAACGCCTTCACCGGAAAAA |
| C3-B-R1 | CM21 | GCTCCGAGTTCTGAGTCTCCGGTCCAATGGACTCACCGGTTCACTCCCACC**T**GATTTCTCGAACCT**C**AAGCTCCT**C**CG**T**AGCCTTTACCTCCAAAACAACGAGTTCAG**T**TCCGAGTTCCC**G**CCGAGTCTACTCAGTCTAACTCGGTTAGTCCGTTTGGATCTCTCTTCCAACGCCTTCACCGGAAAAA |
| C4-B-F1 | RP31 | TGCTGGCATTCATGCTTGAGGTAGCACCCTT**G**ATCCAAGAAACTGAAGCCTCAACTCCGGGAGGCAAATCCACATTGTCAAATTGTGCTTGCATATTCAAATAATCATCGTCATTATCATCATCATTTTCGAACATATAATCGTCATTGTCATCATAATCAGAAATATAAGATCCATCATCATCATTTTCATCTCTGTCATTGCCACCTCCTTCTTTGTCAA |
| C4-B-F1 | W255-02 | TGCTGGCATTCATGCTTGAGGTAGCACCCTT**C**ATCCAAGAAACTGAAGCCTCAACTCCGGGAGGCAAATCCACATTGTCAAATTGTGCTTGCATATTCAAATAATCATCGTCATTATCATCATCATTTTCGAACATATAATCGTCATTGTCATCATAATCAGAAATATAAGATCCATCATCATCATTTTCATCTCTGTCATTGCCACCTCCTTCTTTGTCAA |
| C4-B-F1 | CM21 | TGCTGGCATTCATGCTTGAGGTAGCACCCTT**C**ATCCAAGAAACTGAAGCCTCAACTCCGGGAGGCAAATCCACATTGTCAAATTGTGCTTGCATATTCAAATAATCATCGTCATTATCATCATCATTTTCGAACATATAATCGTCATTGTCATCATAATCAGAAATATAAGATCCATCATCATCATTTTCATCTCTGTCATTGCCACCTCCTTCTTTGTCAA |
| C5-B-R1 | RP31 | TATGCTTCTTGAAGCA**G**TCCTTACAGATTGCTCTTTCGCGAAGAATAAATTGGTAATTGTTACTGGTGATTTTCCTGCACACAATTTTGTTATAATTATCATTTGATTTGTTAGAATACGATGATCCAG**G**TAAAAAAAGTTTTTCCCTAGGGTTTGAAGTCAACTTGTCGCTTAAAAATAGTATCACAACTCATAATTTATCATGTACATGAAAACAAAGTTATGGCTTGGAAGATGGGCGAATGTACATGATAAATTATGAGTTGTGATACTATTTTTAAGCGACAAGTTGACTTCAAACCCTAGGGAAAAACTT |
| C5-B-R1 | W255-02 | TATGCTTCTTGAAGCA**A**TCCTTACAGATTGCTCTTTCGCGAAGAATAAATTGGTAATTGTTACTGGTGATTTTCCTGCACACAATTTTGTTATAATTATCATTTGATTTGTTAGAATACGATGATCCAG**A**TAAAAAAAGTTTTTCCCTAGGGTTTGAAGTCAACTTGTCGCTTAAAAATAGTATCACAACTCATAATTTATCATGTACATGAAAACAAAGTTATGGCTTGGAAGATGGGCGAATGTACATGATAAATTATGAGTTGTGATACTATTTTTAAGCGACAAGTTGACTTCAAACCCTAGGGAAAAACTT |
| C5-B-R1 | CM21 | TATGCTTCTTGAAGCA**A**TCCTTACAGATTGCTCTTTCGCGAAGAATAAATTGGTAATTGTTACTGGTGATTTTCCTGCACACAATTTTGTTATAATTATCATTTGATTTGTTAGAATACGATGATCCAG**A**TAAAAAAAGTTTTTCCCTAGGGTTTGAAGTCAACTTGTCGCTTAAAAATAGTATCACAACTCATAATTTATCATGTACATGAAAACAAAGTTATGGCTTGGAAGATGGGCGAATGTACATGATAAATTATGAGTTGTGATACTATTTTTAAGCGACAAGTTGACTTCAAACCCTAGGGAAAAACTT |
| C6-B-R1 | RP31 | ACTAGACCTTTTACAGTACAAAGCAGTGAACGTAAGGTGAATAAATCTTTGAGCTGCCAATAACTTATCTATTCCGGTGGCCTTTAAGTTTTGCTTTCATTACA**G**ATTAACTATAAC**T**TGTCTACCATAACACGACAATGTATTTTGTTGTGATAGTGGTAACACAGTTTGTTGCTCTTACAATCATAATGTGATTGTGGAANTGTTGAA |
| C6-B-R1 | W255-02 | ACTAGACCTTTTACAGTACAAAGCAGTGAACGTAAGGTGAATAAATCTTTGAGCTGCCAATAACTTATCTA**C**TCCGGTGGCCTTTAAGTTTTGCTTTCATTACA**T**ATTAACTATAACCTGTCTACCATAACACGACAATGTATTTTGTTGTGATAGTGGTAACACAGTTTGTTGCTCTTACAATCATAATGTGATTGTGGAATTGTTGAA |
| C6-B-R1 | CM21 | ACTAGACCTTTTACAGTACAAAGCAGTGAACGTAAGGTGAATAAATCTTTGAGCTGCCAATAACTTATCTA**C**TCCGGTGGCCTTTAAGTTTTGCTTTCATTACA**T**ATTAACTATAACCTGTCTACCATAACACGACAATGTATTTTGTTGTGATAGTGGTAACACAGTTTGTTGCTCTTACAATCATAATGTGATTGTGGAATTGTTGAA |
| C7-B-F1 | RP31 | CAACTGGTTCATTATATGCTGCTG**A**TTTTCTTGACTAATCTTATATGCGTCCACATTCATGAACCGGTGGGTTTGATTTCAAACGTGAAATTTATTA**C**GTACGAATAATACTATTTCGATTTAACGAGACAGAGAATTCAAAAGCAAGTG |
| C7-B-F1 | W255-02 | CAACTGGTTCATTATATGCTGCTG**G**TTTTCTTGACTAATCTTATATGCGTCCACATTCATGAACCGGTGGGTTTGATTTCAAACGTGAAATTTATTA**G**GTACGAATAATACTATTTCGATTTAACGAGACAGAGAATTCAAAAGCAAGTG |
| C7-B-F1 | CM21 | CAACTGGTTCATTATATGCTGCTG**G**TTTTCTTGACTAATCTTATATGCGTCCACATTCATGAACCGGTGGGTTTGATTTCAAACGTGAAATTTATTA**G**GTACGAATAATACTATTTCGATTTAACGAGACAGAGAATTCAAAAGCAAGTG |
| C8-B-F1 | RP31 | CCATCATTTGAAAAGATCCTAGTGCAGCCTGGTG**T**GGACATTA**A**GTTGAATAGTTCCGCAGATGGAATTCT**T**TTACCACA**T**GGTCAGATCAAGAAAGGCAAGAAAGGGTCGACTGCGCTTACCCAAAGTAGGTTCCTCACTCTTGTTGAAA |
| C8-B-F1 | W255-02 | CCATCATTTGAAAAGATCCTAGTGCAGCCTGGTG**A**GGACATTA**T**GTTGAATAGTTCCGCAGATGGAATTCT**C**TTACCACA**CA**GTCAGATCAAGAAAGGCAAGAAAGGGTCGACTGCGCTTACCCAAAGTAGGTTCCTCACTCTTGTTGAAA |
| C8-B-F1 | CM21 | CCATCATTTGAAAAGATCCTAGTGCAGCCTGGTG**A**GGACATTA**T**GTTGAATAGTTCCGCAGATGGAATTCT**C**TTACCACA**CA**GTCAGATCAAGAAAGGCAAGAAAGGGTCGACTGCGCTTACCCAAAGTAGGTTCCTCACTCTTGTTGAAA |
| C9-B-R1 | RP31 | AACTTCCTTTTTGACCA**T**TTGTAAATCTCTACTTA**CGTGCGACCTC**GTTTCGG**T**CAATATATTCCTCCTGGGGAAGGGTCTACTACCTTC**G**TTATCACCTGAGGAAAAATATGACCTGATCAAGGCTTTCT**C**GCTA**C**AAGAGGTTGAAGATGCCCTGGCCGCCACAGATGGCACTAAAGCGCC**A**GGCCCGGATGGTGTTAATGCGGGTTTA |
| C9-B-R1 | W255-02 | AACTTCCTTTTTGACCA**-**TTTTAAATCTCTACTTA**---CTCGA**C**CTC**TTTCGG**A**CAATATATTCCTCCTGGGGAAGGGTCTACTACCTTC**A**TTATCACCTGAGGAAAAATATGACCTGATCAAGGCTTTCT**T**GCTA**A**AAGAGGTTGAAGATGCCCTGGCCGCCACAGATGGCACTAAAGCGCC**G**GGCCCGGATGGTGTTAATGCGGGTTTA |
| C9-B-R1 | CM21 | AACTTCCTTTTTGACCA-TTTTAAATCTCTACTTA-**--CTCGA**C**CTC**TTTCGG**A**CAATATATTCCTCCTGGGGAAGGGTCTACTACCTTC**A**TTATCACCTGAGGAAAAATATGACCTGCAAGGCTTTCT**T**GCTA**A**AAGAGGTTGAAGATGCCCTGGCCGCCACAGATGGCACTAAAGCGCC**G**GGCCCGGATGGTGTTAATGCGGGTTTA |

**Supplementary Table S5.** Statistics of the whole genome sequencing data produced by the DNBseq™ sequencing platform.

| Sample | Total number of raw reads | Total number of clean reads | Total number of mapping reads | Mean depth | Coverage (>=1X) (%) |
| --- | --- | --- | --- | --- | --- |
| WT-1 | 152,770,516 | 151,401,132 | 149,548,337 | 51.83 | 87.0 |
| WT-2 | 147,140,934 | 145,428,020 | 143,176,214 | 49.76 | 86.5 |
| WT-3 | 116,718,896 | 115,555,418 | 113,649,683 | 39.51 | 86.9 |
| WT-4 | 103,532,222 | 102,344,872 | 100,703,692 | 35.06 | 86.6 |
| CM21 | 122,301,078 | 121,010,602 | 119,028,080 | 41.21 | 83.2 |
| CM23 | 118,003,120 | 116,748,544 | 114,912,796 | 39.84 | 86.5 |
| Average | 135,386,818 | 134,074,838 | 132,230,566 | 45.83 | 86.8 |

**Supplementary Table S6.** Median propidium iodide (PI) fluorescence values (YL2 median) for each sample analyzed by flow cytometry in Figure 5.

| Sample(s) | Peak | YL2 median |
| --- | --- | --- |
| WT only | WT | 414,248 |
| CM21 only | CM21 | 834,156 |
| WT + CM21 | WT | 385,209 |
| WT + CM21 | CM21 | 783,419 |
| WT only | WT | 683,230 |
| CM23 only | CM23 | 631,170 |
| WT + CM23 | WT | n/a |
| WT + CM23 | CM23 | n/a |
