# Supplementary material for: Chromosome-level changes and genome elimination by manipulation of CENH3 in carrot (Daucus carota)
Source: Front Plant Sci. 2023 Nov 15;14:1294551. doi: 10.3389/fpls.2023.1294551 (PMC10684906; doi:10.3389/fpls.2023.1294551)
Supplement: Supplementary file 2 [file DataSheet_1.pdf]

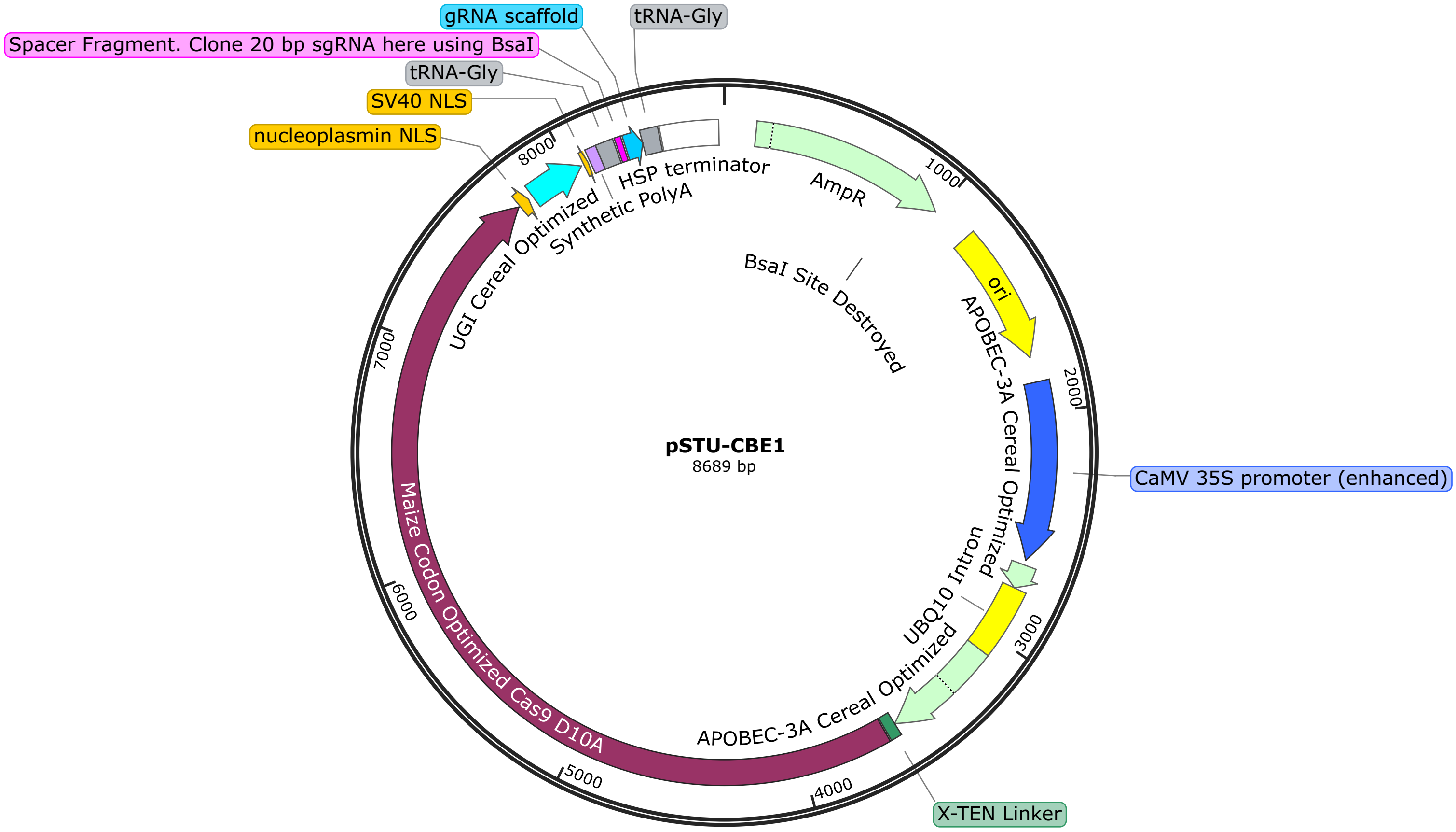
gRNA scaffold
tRNA-Gly
Spacer Fragment. Clone 20 bp sgRNA here using BsaI
tRNA-Gly
SV40 NLS
nucleoplasmin NLS
8000
HSP terminator
Synthetic PolyA
AmpR
1000
UGI Cereal Optimized
BsaI Site Destroyed
ori
APOBEC-3A Cereal Optimized
7000
2000
pSTU-CBE1
8689 bp
CaMV 35S promoter (enhanced)
Maize Codon Optimized Cas9 D10A
UBQ10 Intron
6000
3000
APOBEC-3A Cereal Optimized
5000
4000
X-TEN Linker

STU-CBE1 Plasmid Sequence

gacgtcaggtggcacttttcggggaaatgtgcgcggaacccctatttgtttatttttctaaatacattcaaatatgtatccgctcatgagacaataaccctgataaa
tgcttcaataatattgaaaaaggaagagtatgagtattcaacatttccgtgtcgcccttattcccttttttgcggcattttgccttcctgtttttgctcacccagaaacg
ctggtgaaagtaaaagatgctgaagatcagttgggtgcacgagtgggttacatcgaactggatctcaacagcggtaagatccttgagagttttcgccccgaaga
acgttttccaatgatgagcacttttaaagttctgctatgtggcgcggtattatcccgtattgacgccgggcaagagcaactcggtcgccgcatacactattctcaga
atgacttggttgagtactcaccagtcacagaaaagcatcttacggatggcatgacagtaagagaattatgcagtgctgccataaccatgagtgataacactgcg
gccaacttacttctgacaacgatcggaggaccgaaggagctaaccgcttttttgcacaacatgggggatcatgtaactcgccttgatcgttgggaaccggagctg
aatgaagccataccaaacgacgagcgtgacaccacgatgcctgtagcaatggcaacaacgttgcgcaaactattaactggcgaactacttactctagcttcccg
gcaacaattaatagactggatggaggcggataaagttgcaggaccacttctgcgctcggcccttccggctggctggtttattgctgataaatctggagccggtga
gcgtggatctcgcggtatcattgcagcactggggccagatggtaagccctcccgtatcgtagttatctacacgacggggagtcaggcaactatggatgaacgaa
atagacagatcgctgagataggtgcctcactgattaagcattggtaactgtcagaccaagtttactcatatatactttagattgatttaaaacttcatttttaatttaa
aaggatctaggtgaagatcctttttgataatctcatgaccaaaatcccttaacgtgagttttcgttccactgagcgtcagaccccgtagaaaagatcaaaggatct
tcttgagatcctttttttctgcgcgtaatctgctgcttgcaaacaaaaaaaccaccgctaccagcggtggtttgtttgccggatcaagagctaccaactctttttccg
aaggtaactggcttcagcagagcgcagataccaaatactgttcttctagtgtagccgtagttaggccaccacttcaagaactctgtagcaccgcctacatacctc
gctctgctaatcctgttaccagtggctgctgccagtggcgataagtcgtgtcttaccgggttggactcaagacgatagttaccggataaggcgcagcggtcgggc
tgaacggggggttcgtgcacacagcccagcttggagcgaacgacctacaccgaactgagatacctacagcgtgagctatgagaaagcgccacgcttcccgaa
gggagaaaggcggacaggtatccggtaagcggcagggtcggaacaggagagcgcacgagggagcttccagggggaaacgcctggtatctttatagtcctgt
cgggtttcgccacctctgacttgagcgtcgatttttgtgatgctcgtcaggggggcggagcctatggaaaaacgccagcaacgcggcctttttacggttcctggcc
ttttgctggccttttgctcacatgttctttccgatgtttaaaccctgcaggattggctagagcagcttgccaacatggtggagcacgacactctcgtctactccaaga
atatcaaagatacagtctcagaagaccaaagggctattgagacttttcaacaaagggtaatatcgggaaacctcctcggattccattgcccagctatctgtcact
tcatcaaaaggacagtagaaaaggaaggtggcacctacaaatgccatcattgcgataaaggaaaggctatcgttcaagatgcctctgccgacagtggtccca
aagatggacccccacccacgaggagcatcgtggaaaaagaagacgttccaaccacgtcttcaaagcaagtggattgatgtgataacatggtggagcacgaca
ctctcgtctactccaagaatatcaaagatacagtctcagaagaccaaagggctattgagacttttcaacaaagggtaatatcgggaaacctcctcggattccatt
gcccagctatctgtcacttcatcaaaaggacagtagaaaaggaaggtggcacctacaaatgccatcattgcgataaaggaaaggctatcgttcaagatgcctct
gccgacagtggtcccaaagatggacccccacccacgaggagcatcgtggaaaaagaagacgttccaaccacgtcttcaaagcaagtggattgatgtgatatct
ccactgacgtaagggatgacgcacaatcccactatccttcgcaagaccttcctctatataaggaagttcatttcatttggagaggacacgctgaaatcaccagtct
ctctctacaaatctatctctctcgagtctaccgaattcggcatggaggccagcccggctagcggcccaaggcatctcatggacccgcacatcttcaccagcaact
tcaacaacggcatcggcaggcacaagacctacttgtgctacgaggtaagtttctgtgttccttattctctcaaaatcttcgatttgttttcgttcgatcccaatttcgt
atatgttctttggtttagattctgttaatcttagatcgaagacgattttctgggtttgatcgttagatatcatcttaattctcgattagggtttcatagatatcatccgatt
tgttcaaataatttgagttttgtcgaataattactcttcgatttgtgatttctatctagatctggtgttagtttctagtttgtgcgatcgaatttgtcgattaatctgagttt
ttctgattaacaggtggagaggctcgacaacggaacctccgtgaagatggaccaacacagggggttcctccacaaccaagccaagaacctcctctgcggcttc
tacggcaggcacgccgagttgaggttcctcgacttggtgccatccctccaactcgatccagcccaaatctaccgcgtgacctggttcatctcctggtccccatgctt
ctcctggggttgcgccggcgaggttcgggctttcctccaagaaaacacccacgtccgcctccgcattttcgccgccaggatctatgattacgaccctctctacaag
gaggccctccagatgctgcgggacgccggtgctcaggtgagtatcatgacctacgacgagttcaagcactgctgggacaccttcgttgaccaccagggctgccc
attccaaccatgggacggtctggatgaacacagccaagccttgtccggcaggctccgggccatcctccaaaaccaggggaactccgggagcgagacgccagg
cacctccgagtcggccaccccagaatctggtaccgacaagaagtacagcatcggcctggccattgggactaactctgttggctgggccgtgatcaccgacgagt
acaaggtgccctcaaagaagttcaaggtcctgggcaacaccgatcggcattccatcaagaagaatctcattggcgctctcctgttcgacagcggcgagacggct
gaggctacgcggctcaagcgcaccgcccgcaggcggtacacgcgcaggaagaatcgcatctgctacctccaggagattttctccaacgagatggcgaaggttg
acgattctttcttccacaggctggaggagtcattcctcgtggaggaggataagaagcacgagcggcatccaatcttcggcaacattgtcgacgaggttgcctacc
acgagaagtaccctacgatctaccatctgcggaagaagctcgtggactccacagataaggcggacctccgcctgatctacctcgctctggcccacatgattaag
ttcaggggccatttcctgatcgagggggatctcaacccggacaatagcgatgttgacaagctgttcatccagctcgtgcagacgtacaaccagctcttcgaggag
aaccccattaatgcgtcaggcgtcgacgcgaaggctatcctgtccgctaggctctcgaagtctcggcgcctcgagaacctgatcgcccagctgccgggcgagaa
gaagaacggcctgttcgggaatctcattgcgctcagcctggggctcacgcccaacttcaagtcgaatttcgatctcgctgaggacgccaagctgcagctctccaa
ggacacatacgacgatgacctggataacctcctggcccagatcggcgatcagtacgcggacctgttcctcgctgccaagaatctgtcggacgccatcctcctgtc
tgatattctcagggtgaacaccgagattacgaaggctccgctctcagcctccatgatcaagcgctacgacgagcaccatcaggatctgaccctcctgaaggcgc
tggtcaggcagcagctccccgagaagtacaaggagatcttcttcgatcagtcgaagaacggctacgctgggtacattgacggcggggcctctcaggaggagtt
ctacaagttcatcaagccgattctggagaagatggacggcacggaggagctgctggtgaagctcaatcgcgaggacctcctgaggaagcagcggacattcgat
aacggcagcatcccacaccagattcatctcggggagctgcacgctatcctgaggaggcaggaggacttctaccctttcctcaaggataaccgcgagaagatcg
agaagattctgactttcaggatcccgtactacgtcggcccactcgctaggggcaactcccgcttcgcttggatgacccgcaagtcagaggagacgatcacgccg

tggaacttcgaggaggtggtcgacaagggcgctagcgctcagtcgttcatcgagaggatgacgaatttcgacaagaacctgccaaatgagaaggtgctcccta
agcactcgctcctgtacgagtacttcacagtctacaacgagctgactaaggtgaagtatgtgaccgagggcatgaggaagccggctttcctgtctggggagcag
aagaaggccatcgtggacctcctgttcaagaccaaccggaaggtcacggttaagcagctcaaggaggactacttcaagaagattgagtgcttcgattcggtcga
gatctctggcgttgaggaccgcttcaacgcctccctggggacctaccacgatctcctgaagatcattaaggataaggacttcctggacaacgaggagaatgagg
atatcctcgaggacattgtgctgacactcactctgttcgaggaccgggagatgatcgaggagcgcctgaagacttacgcccatctcttcgatgacaaggtcatga
agcagctcaagaggaggaggtacaccggctgggggaggctgagcaggaagctcatcaacggcattcgggacaagcagtccgggaagacgatcctcgacttc
ctgaagagcgatggcttcgcgaaccgcaatttcatgcagctgattcacgatgacagcctcacattcaaggaggatatccagaaggctcaggtgagcggccagg
gggactcgctgcacgagcatatcgcgaacctcgctggctcgccagctatcaagaaggggattctgcagaccgtgaaggttgtggacgagctggtgaaggtcat
gggcaggcacaagcctgagaacatcgtcattgagatggcccgggagaatcagaccacgcagaagggccagaagaactcacgcgagaggatgaagaggatc
gaggagggcattaaggagctggggtcccagatcctcaaggagcacccggtggagaacacgcagctgcagaatgagaagctctacctgtactacctccagaat
ggccgcgatatgtatgtggaccaggagctggatattaacaggctcagcgattacgacgtcgatcatatcgttccacagtcattcctgaaggatgactccattgac
aacaaggtcctcaccaggtcggacaagaaccggggcaagtctgataatgttccttcagaggaggtcgttaagaagatgaagaactactggcgccagctcctga
atgccaagctgatcacgcagcggaagttcgataacctcacaaaggctgagaggggcgggctctctgagctggacaaggcgggcttcatcaagaggcagctgg
tcgagacacggcagatcactaagcacgttgcgcagattctcgactcacggatgaacactaagtacgatgagaatgacaagctgatccgcgaggtgaaggtcat
caccctgaagtcaaagctcgtctccgacttcaggaaggatttccagttctacaaggttcgggagatcaacaattaccaccatgcccatgacgcgtacctgaacgc
ggtggtcggcacagctctgatcaagaagtacccaaagctcgagagcgagttcgtgtacggggactacaaggtttacgatgtgaggaagatgatcgccaagtcg
gagcaggagattggcaaggctaccgccaagtacttcttctactctaacattatgaatttcttcaagacagagatcactctggccaatggcgagatccggaagcgc
cccctcatcgagacgaacggcgagacgggggagatcgtgtgggacaagggcagggatttcgcgaccgtcaggaaggttctctccatgccacaagtgaatatc
gtcaagaagacagaggtccagactggcgggttctctaaggagtcaattctgcctaagcggaacagcgacaagctcatcgcccgcaagaaggactgggatccg
aagaagtacggcgggttcgacagccccactgtggcctactcggtcctggttgtggcgaaggttgagaagggcaagtccaagaagctcaagagcgtgaaggag
ctgctggggatcacgattatggagcgctccagcttcgagaagaacccgatcgatttcctggaggcgaagggctacaaggaggtgaagaaggacctgatcatta
agctccccaagtactcactcttcgagctggagaacggcaggaagcggatgctggcttccgctggcgagctgcagaaggggaacgagctggctctgccgtccaa
gtatgtgaacttcctctacctggcctcccactacgagaagctcaagggcagccccgaggacaacgagcagaagcagctgttcgtcgagcagcacaagcattac
ctcgacgagatcattgagcagatttccgagttctccaagcgcgtgatcctggccgacgcgaatctggataaggtcctctccgcgtacaacaagcaccgcgacaa
gccaatcagggagcaggctgagaatatcattcatctcttcaccctgacgaacctcggcgcccctgctgctttcaagtacttcgacacaactatcgatcgcaagag
gtacacaagcactaaggaggtcctggacgcgaccctcatccaccagtcgattaccggcctctacgagacgcgcatcgacctgtctcagctcgggggcgacact
agtaagaggcccgcagcaaccaagaaggcagggcaagcaaagaagaagaagacgcgtgactccggcggcagcaccaacctgtccgacatcatcgagaag
gagacgggcaagcaactcgtgatccaggagagcatcctcatgctgccagaggaggtggaggaggtcatcggcaacaagccagagtccgacatcctggtgcac
accgcctacgacgagtccaccgacgagaacgtcatgctcctgaccagcgacgccccagagtacaagccatgggccctcgtcatccaggacagcaacggggag
aacaagatcaagatgctgtcgggggggagcccaaagaagaagcggaaggtgtaggagctcaaaaaaaaaaaaaaaaaaaaaaaaaaaaaaaaaaaaa
aaaaaaaaaacaaagcaccagtggtctagtggtagaatagtaccctgccacggtacagacccgggttcgattcccggctggtgcaggagacccagttgc
gcagcctgaatggcgaatggggtctcagttttagagctagaaatagcaagttaaaataaggctagtccgttatcaacttgaaaaagtggcaccgagtcggtgca
acaaagcaccagtggtctagtggtagaatagtaccctgccacggtacagacccgggttcgattcccggctggtgcaggatccatatgaagatgaagatgaaat
atttggtgtgtcaaataaaaagcttgtgtgcttaagtttgtgtttttttcttggcttgttgtgttatgaatttgtggctttttctaatattaaatgaatgtaagatcacatt
ataatgaataaacaaatgtttctataatccattgtgaatgttttgttggatctcttctgcagcatataactactgtatgtgctatggtatggactatggaatatgatta
aagataagcgcgcgcggtttaaacatcattgg
